# Exploring whole proteome to contrive multi-epitope-based vaccine for NeoCoV: An immunoinformtics and *in-silico* approach

**DOI:** 10.3389/fimmu.2022.956776

**Published:** 2022-08-03

**Authors:** Shahkaar Aziz, Muhammad Waqas, Sobia Ahsan Halim, Amjad Ali, Aqib Iqbal, Maaz Iqbal, Ajmal Khan, Ahmed Al-Harrasi

**Affiliations:** ^1^ Institute of Biotechnology and Genetic Engineering, the University of Agriculture Peshawar, Peshawar, Pakistan; ^2^ Natural and Medical Sciences Research Center, University of Nizwa, Birkat-ul-Mouz, Nizwa, Oman; ^3^ Department of Biotechnology and Genetic Engineering, Hazara University Mansehra, Mansehra, Pakistan

**Keywords:** immunoinformatics, multi-epitope vaccine, subunit vaccine, epitopes prediction, vaccine design, NeoCoV

## Abstract

Neo-Coronavirus (NeoCoV) is a novel Betacoronavirus (β-CoVs or Beta-CoVs) discovered in bat specimens in South Africa during 2011. The viral sequence is highly similar to Middle East Respiratory Syndrome, particularly that of structural proteins. Thus, scientists have emphasized the threat posed by NeoCoV associated with human angiotensin-converting enzyme 2 (ACE2) usage, which could lead to a high death rate and faster transmission rate in humans. The development of a NeoCoV vaccine could provide a promising option for the future control of the virus in case of human infection. *In silico* predictions can decrease the number of experiments required, making the immunoinformatics approaches cost-effective and convenient. Herein, with the aid of immunoinformatics and reverse vaccinology, we aimed to formulate a multi-epitope vaccine that may be used to prevent and treat NeoCoV infection. Based on the NeoCoV proteins, B-cell, cytotoxic T lymphocyte (CTL), and helper T lymphocyte (HTL) epitopes were shortlisted. Four vaccines (Neo-1–4) were devised by fusing shortlisted epitopes with appropriate adjuvants and linkers. The secondary and three-dimensional structures of final vaccines were then predicted. The binding interactions of these potential vaccines with toll-like immune receptors (TLR-2, TLR-3, and TLR-4) and major histocompatibility complex molecules (MHC-I and II) reveal that they properly fit into the receptors’ binding domains. Besides, Neo-1 and Neo-4 vaccines exhibited better docking energies of -101.08 kcal/mol and -114.47 kcal/mol, respectively, with TLR-3 as compared to other vaccine constructs. The constructed vaccines are highly antigenic, non-allergenic, soluble, non-toxic, and topologically assessable with good physiochemical characteristics. Codon optimization and *in-silico* cloning confirmed efficient expression of the designed vaccines in *Escherichia coli* strain K12. *In-silico* immune simulation indicated that Neo-1 and Neo-4 vaccines could induce a strong immune response against NeoCoV. Lastly, the binding stability and strong binding affinity of Neo-1 and Neo-4 with TLR-3 receptor were validated using molecular dynamics simulations and free energy calculations (Molecular Mechanics/Generalized Born Surface Area method). The final vaccines require experimental validation to establish their safety and effectiveness in preventing NeoCoV infections.

## 1 Introduction

Coronaviruses (CoVs) are an enveloped positive-stranded RNA virus family divided into four genera, including α–, β–, γ–, and δ–CoV. The first two genera can infect mammals (bats and humans), whereas the latter primarily infect birds and, sometimes, mammals. Most coronaviruses that infect humans are believed to have originated in bats—the key mammalian coronavirus reservoir (1, 2). Bat-CoVs have received particular attention since several recently seen CoVs have been associated with unexpected disease outbreaks in the present century, causing high fatality rates and significant economic impact. Three such viruses suggested to be transmitted from bats to humans include Severe Acute Respiratory Syndrome Coronavirus (SARS-CoV), Middle East Respiratory Syndrome Coronavirus (MERS-CoV), and the latest Severe Acute Respiratory Syndrome Coronavirus-2 (SARS-CoV-2) (2, 3).

MERS-CoV belongs to the C lineage of the Beta-CoV (Merbecoviruses), which offers a significant risk due to its high fatality rate of over 35%. Several animals, such as bats, hedgehogs, and camels, carry merbecoviruses indefinitely. Although camels are known as the intermediate hosts of MERS-CoV, bats, particularly those belonging to the Vespertilionidae family, are primarily thought to represent the virus’s evolutionary source or intermediate ancestor (4). Neo-Coronavirus (NeoCoV), a novel Beta-CoV, was discovered in a Neoromicia cf. zuluensis bat specimen in 2011. This virus varied from MERS-CoV by single amino acid substitution (0.3%) in the RdRp gene fragment (translated 816-nt) and by amino acid sequence distance of 10.9% in the glycoprotein coding gene enables attachment and entry of CoV into the cell. Therefore, NeoCoV and MERS-CoV are more closely related to each other. Victor Max Corman and colleagues suggested 85% sequence similarity between these two CoVs, indicating that NeoCoV and MERS-CoV originated from common viral species (5).

Scientists have emphasized the threat posed by NeoCoV in bats reported in South Africa, which could have a high death rate and faster spread, amidst the control of cases resurging due to many evolving variants of Severe acute respiratory syndrome coronavirus 2 (SARS-CoV-2) (6). It has also been proposed that if the NeoCoV attains mutations and causes human infection, it might develop into Coronavirus disease-22 (COVID-22) and cause symptoms three times as severe as COVID-19. Any outbreak caused by this viral strain, may trigger a 30% increase in fatalities, comparable to MERS. Around 17 million people could die due to COVID-22, compared to COVID-19, which has resulted in over 6 million people succumbing to death until now (6, 7). Nevertheless, it is worth noting that NeoCoV has yet to be confirmed in people and no reported human fatalities has observed with this virus. At the same time, the globe deals with the coronavirus disease-2019 pandemic. In Jan 2022, World Health Organization (WHO) warned about NeoCoV and demanded further research to determine NeoCoV’s possible threat to humans (6).

MERS-CoV and several related bat-coronaviruses utilize human dipeptidyl peptidase-4 (DPP4), a functional receptor located on the airways cell surface (i.e., lungs), as an entry receptor (8–10). Nonetheless, the cell entry receptor for NeoCoV is unspecified so far (11). NeoCoV and closely related PDF-2180-CoV can use certain forms of bat angiotensin-converting enzyme 2 (ACE2) and human ACE2 for cell entry, according to the latest research in preprint (2). The NeoCoV virus utilizes its S1 subunit at carboxyl-terminal domains (S1-CTD) of spike protein to bind with ACE2 with great affinity and species specificity. Besides, a molecular determinate (Asp338) was uncovered at the binding interface of the virus that prevent NeoCoV entry *via* human ACE2 (2). Researchers found that T510F mutation at the receptor-binding motif leads to increase the efficiency of NeoCoV to infect the human cells expressing ACE2 receptor (2). Furthermore, antibodies produced by natural infection or vaccination against SARS-CoV-2 or MERS-CoV are incapable of neutralizing NeoCoV infection. Also, the potential use of ACE-2 receptor by MERS-related viruses has been indicated for the first time, underlining a possible health threat posed by “MERS-CoV-2” with high mortality and spread rate (2).

Presently, there is limited knowledge regarding NeoCoV, and it is uncertain if the virus can be transmitted to humans or spread worldwide. Further research can help understand this new coronavirus and its immunology and vaccinations if it occurs in humans. Moreover, the COVID-19 pandemic is still ongoing. Thus, there is an urgent need to improve vaccination rates in both human and animals (12) and oversee other potential health implications, including the NeoCoV.

Vaccination is a crucial strategy for viral control and eradication (13). The development of a NeoCoV vaccine could provide a promising option for the future control of this virus if it infects humans. Conventional vaccine development procedures take a long time and require a great deal of manual effort (14). Immunoinformatics tools evaluate the host immune response to provide alternative techniques in order to formulate economical and advantageous vaccines against the diseases since predictions can curtail the number of *in vitro* tests required (15, 16). Vaccines based on the structural and non-structural proteins (NSPs) are reported to induce protective immune responses (17, 18). Scientifically rigorous approaches based on various proteins have been exploited to design multi-epitope subunits for the viral and parasite diseases, including malaria and SARS-CoV-2 (13, 19, 20). Here, we applied immunoinformatics techniques to predict numerous immunogenic proteins from the whole proteome of NeoCoV and developed a multi-epitope vaccine in this research by studying the structural and NSPs of NeoCoV.

## 2 Materials and method

### 2.1 Retrieval of protein sequence

The complete amino acid sequence of NeoCoV proteins was retrieved from the National Center for Biotechnology Information (NCBI) database (https://www.ncbi.nlm.nih.gov/) in FASTA format. These comprise four structural proteins and 19 non-structural protein components. The retrieved proteins and their NCBI accession numbers are provided in the **Supplementary Table S1**.

### 2.2 Selection of protein for vaccine formulation

The position and residue range of the viral protein sequences and vaccine constructs were determined using the TMHMM-2.0 prediction tool (21). The localization predictions of the viral proteins were performed by DeepLoc (22) which uses a template-free algorithm that implements a deep neural network to envisage subcellular localization of proteins with acceptable accuracy using only sequence information (22).

### 2.3 Allergenicity, antigenicity, and toxicity prediction

Owing to their significance in food and/or food products, allergenicity prediction is an essential step in medications and biopharmaceuticals (23). The AllerTOP v2.0 (24) and AllergenFP P1.0 (25) were used to predict the allergenicity of the viral protein and epitopes. The former employs E-descriptors and an auto cross-covariance (ACC) transformation (26). The latter is a binary classifier between allergen and non-allergen that transforms protein sequences into uniform vectors of equal length using an ACC transformation, as described by Dimitrov et al. (24). The antigenicity of the viral proteins, epitopes, and vaccine construct was determined using a VaxiJen tool (27). The amino acid sequence of the query was used as an input, with the chosen organism target of ‘virus’ at a cut-off of 0.4. ANTIGENpro (http://scratch.proteomics.ics.uci.edu/) was employed to confirm vaccine constructs antigenicity. Using the Toxinpred server (28), safety evaluation of viral proteins and epitopes were performed.

### 2.4 B-cell epitopes prediction

For the primary prediction of linear B-cell epitopes, the BCPreds server (29) was used. This server employs a kernel approach (30) with 75% sensitivity; the epitope length was fixed at 20 amino acids. It has been found that using various techniques to predict epitopes improves the probability of true positives in epitope prediction (31). Thus, the ABCpred (32) and BepiPred-2.0 (33) were employed for validation. Epitope scores of ABCpred were calculated using a threshold of 0.5 and a window length of 20. Further, leveraging the Ellipro server (34) with default parameters, the improved modelled structure of the multi-epitope vaccine was submitted to confirmational B-cell epitopes prediction.

### 2.5 MHC class-I binding epitopes prediction

**Figure 1:**
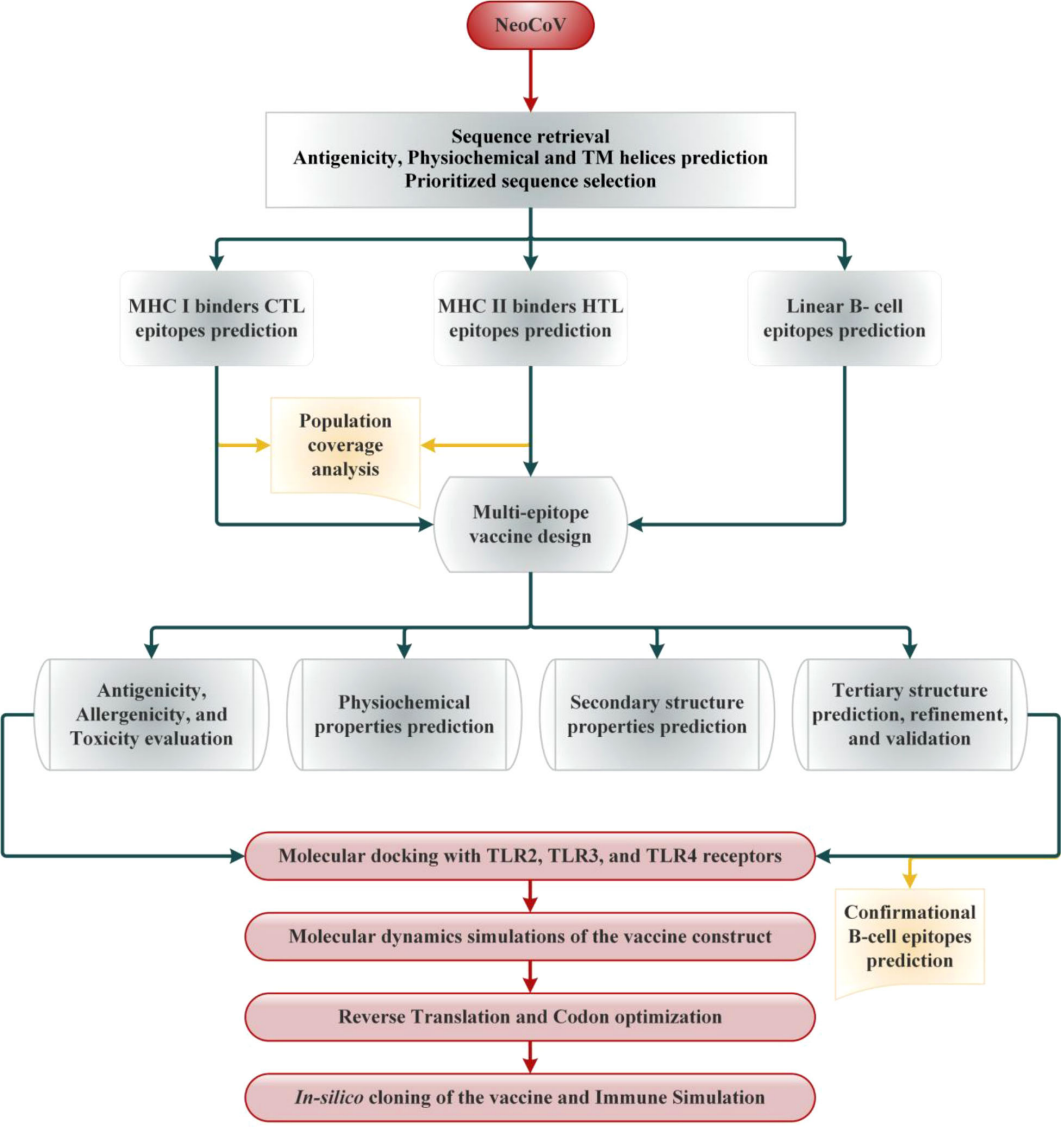
Schematic illustration of the overall strategy implemented in this study to design multi-epitope-based vaccine from NeoCoV whole proteome.

Selected viral proteins were subjected to MHC-I epitopes prediction employing the NetCTL 1.2 server. The binding affinity of the predicted class epitopes was tested against 12 different HLA supertypes, including A1, A2, A3, A24, A26, B7, B8, B27, B39, B44, B58, and B62 (35). Peptides were ranked based on a combined score; all other parameters were set as default. Only those epitopes were subjected to downstream analysis at this stage that indicated binding with at least four HLA I supertypes (36). To predict the immunogenicity of shortlisted MHC-I epitopes, the online bioinformatics server IEDB was used (36). The epitopes showing positive immunogenicity scores were chosen for further analysis. Then, predicted MHCs epitopes class were also tested to find if they are antigenic, non-allergen and safe. The IEDB SMM method (http://tools.iedb.org/mhcii/) was used to test the binding affinity of shortlisted epitopes with their respective MHC-I alleles by predicting the IC_50_ values. The IC_50_ threshold value of 500nM as an MHC affinity specifies the significant immunogenicity for MHC-I restricted T cells (37). The prediction performance of the NetCTL 1.2 server was confirmed using the CTLPred server (38) with a combined approach and default setup (ANN and SVM thresholds of 0.51 and 0.36). The methodology of the vaccine selection and construction is illustrated in **Figure 1**.

### 2.6 MHC class-II binding epitopes prediction

NetMHCIIpan was used to predict the MHC-II binding epitopes from the full-length sequence of selected viral proteins. DRB10101, DRB10301, DRB10401, DRB10701, DRB10801, DRB11101, DRB11301, and DRB1 1501 were the HLA II supertype alleles targeted for epitope searching [30]. These HLA II supertype alleles cover 95% of the world’s HLA variations (39). We employed NetMHCIIpan because it is presently the most reliable MHC-II epitope prediction (40). First, the predicted helper T lymphocyte (HTL) epitopes that interacted with at least three supertypes alleles were evaluated for antigenicity, non-allergenicity, and non-toxicity. Following that, B-cell, and MHC-I overlapping peptides were removed. The resulting epitopes were prioritized using the IEDB SMM method with an IC_50_ cut-off of 500 nM. Interferon-gamma (IFN-γ) production is critical for viral clearance and the activation of the host immune response. Using the IFN epitope server (http://crdd.osdd.net/raghava/ifnepitope/), we predicted the IFN-γ induction efficiency of selected MHC-II epitopes. Furthermore, the IL4pred server (https://webs.iiitd.edu.in/raghava/il4pred/) and IL10pred server (https://webs.iiitd.edu.in/raghava/il10pred/) were used to assess the interleukin-4 (IL-4; with a threshold of 0.2) and interleukin-10 (IL-10; with a threshold of 0.3) secretion potential of selected MHC-II epitopes, respectively. Finally, the prioritized epitopes sequences were submitted to the MHCPred server (41) in order to determine their 9-mer peptide by testing the binding affinity with the most common allele, DRB10101.

### 2.7 Population coverage analysis

Ethnicity and geography influence the distribution and expression of different HLA alleles (42). It can influence the formulation of an effective epitope-based vaccine. Therefore, IEDB (http://tools.iedb.org/population/) population coverage tool was used to estimate the vaccine’s coverage in the target population. For that purpose, we separately and collectively assessed the picked CTL and HTL epitopes from class I and II MHC and their HLA binding alleles (43). Herein, we focused on the whole global coverage of the alleles and regions of many continents.

### 2.8 Evaluation of human homology and epitopes novelty

To decrease the cross-reactivity in host cells, nonoverlapping epitopes and vaccine constructs were BLASTp against human proteome (taxon id: 9606). The IEDB server (https://www.iedb.org/) was used to determine whether the prioritized epitopes had already been experimentally tested in previous investigations.

### 2.9 Vaccine designing

Prioritized B and T-cell epitopes from NeoCoV whole proteome were chosen for the final vaccine formulation. As safe vaccination adjuvants, compounds with immunomodulatory capabilities were added to the vaccine constructions to boost the immune system. To attach the adjuvants to the epitopes, the rigid linker EAAAK was used. The GPGPG linkers, rich in glycine and proline, were employed to space the MHC-II and B-cell epitopes. AAY, an efficient and flexible linker, was also used to connect the MHC-I epitopes. In addition, the PADRE (Pan DR T Helper Epitope) sequence was inserted to improve the vaccine construct immunogenicity. Using three adjuvants (β-defensin, heparin-binding hemagglutinin (HBHA), and 50S ribosomal protein L7/L12), four vaccines (Neo-1 to Neo-4) were prepared (44).

### 2.10 Physiochemical properties, solubility, and toxicity analysis

The physicochemical characteristics of the final vaccine and its subunits (epitopes) were estimated through ExPASy ProtParam server (45). The solubility of vaccine constructs was assessed using the SOLpro (46) and Protein-sol (47) servers. SOLpro uses SVM-based technique to predict the protein sequence solubility, with a tenfold cross-validation-estimated overall accuracy of about 74% (48). Protein-sol is based on the data of protein solubility in an *Escherichia coli* expression system (49). Using the ToxinPred server (28), the toxicity of the final vaccine constructs and each of its subunits was predicted. To distinguish between toxic and non-toxic, ToxinPred uses an SVM model based on a collection of 1805 toxic peptides (50).

### 2.11 Secondary structure prediction

The primary sequence of vaccines was deployed on NetSurfP-3.0 server (https://services.healthtech.dtu.dk/service.php?NetSurfP-3.0) to predict the secondary structure features of the constructed vaccines. To generate sequence embedding, this server exploits the ESM-1b language model, which is then processed through deep neural network. This server was also used to predict vaccine constructs solvent accessibility and disorder regions.

### 2.12 Three-dimensional structure modeling and refinement of vaccine

The RoseTTAFold tool (51) was employed to predict the 3-dimensional (3D) structure of the multi-epitope vaccine. Then, the predicted 3D model was submitted for structural refinement to the GalaxyRefine server (52). Using molecular dynamics simulation, GalaxyRefine carries out the correction of side chains and stabilizes the structure (53). The improved model’s quality was assessed using the GDT-HA score, Molprobity score, clash score, RMSD score, and Ramachandran plot score. Finally, using ERRAT and ProSAweb servers, validation of our refined 3D model of vaccine construct was conducted (54, 55).

### 2.13 Molecular docking

MOE2020 (56) software was used for modeled vaccine-immune receptor docking using a protein-protein docking protocol of this software. A rigid body refinement method was applied, and the final 30 poses were retained. We chose several toll-like immune receptors (TLR) for docking, such as TLR2 (PDB ID: 2Z7X [https://www.rcsb.org/structure/2Z7X]), TLR3 (PDB ID: 2A0Z [https://www.rcsb.org/structure/2A0Z]), and TLR4 (PDB ID: 3FXI [https://www.rcsb.org/structure/3FXI]) and major histocompatibility complex (MHC) I (PDB ID: 1AKJ [https://www.rcsb.org/structure/1AKJ]) and II (PDB ID: 3L6F [https://www.rcsb.org/structure/3L6F]). Based on the lowest docking energy (high binding affinity) score, top docked complexes were subjected to further analysis. For the 2D interaction analysis, PDBsum web server was used (57) and Blender software (58) was used to illustrate the 3D structure of the docked complexes.

### 2.14 Optimization of codon and *in-silico* cloning

The residues sequence of the finalized vaccine was used as an input in EMBOSS Backtranseq (https://www.ebi.ac.uk/Tools/st/emboss_backtranseq/) to obtain the cDNA of our constructs. The vaccine construct was then codon optimized using the Java Codon Adaptation Tool (JCat) service (59). This server provides results in terms of percent GC content and codon adaptation index (CAI), which were observed to assess the expression potential of protein. Also, at the N- and C-terminus of the vaccine codon sequence, cleavage sites for *Xho*I and *Nde*I enzymes were added. Using the SnapGene program (https://www.snapgene.com/), the optimized multi-epitope vaccine construct sequence was cloned between the *Xho*I and *Nde*I loci in the expression vector, pET28a (+).

### 2.15 Computational immune simulation

A computational immunological simulation was conducted *via* C-ImmSim server to assess the developed vaccine immunogenicity (60). This server employs machine learning algorithms and a position-specific scoring matrix (PSSM) to predict epitope and assess immunological interactivities (60). The immunological simulation was carried out according to the procedure described elsewhere (61). For 1050 simulation steps (about 12 months), three in-silico doses were given at suggested 4-week intervals and at time steps of 1, 84, and 170 (one time step is 8 hours of everyday living). The default settings were kept for all other triggering parameters.

### 2.16 Simulation study of the vaccine-TLRs complex

Molecular dynamics (MD) simulations of the multi-epitope vaccine/TLR complexes were performed using AMBER 20 (62) software with the ff19SB forcefield (63). Each system was initially solvated in a truncated octahedral box with 10Å buffer and an OPC water model before being neutralized with Na+ and Cl^-^ ions (64). The SHAKE method was used to limit all covalent bonds with hydrogen (65). The particle-mesh Ewald method (66) with a cut-off of 8Å was used to calculate long-range electrostatics. Using the PMEMD engine (67) on GPUs, parallel scaling in long-range electrostatics was enhanced. A continuum model was used to calculate the van der Waals long-range interactions. The LEaP module was also utilized to help find missing hydrogen atoms. Following these preparations, the complex systems were energy minimized in two stages (2000 steps steepest descent minimization; 10,000 steps conjugate gradients minimization) (68). In a microcanonical ensemble (NVE), each system was then heated from 0.1 to 300K in 400ps. A Langevin thermostat (69) and a collision frequency of 2.0ps^-1^ were used to control the kinetic energy of harmonic oscillators for dynamic propagation. The density was then changed using the same way in the 400ps run. In an NVE ensemble with no restriction and a pressure relaxation duration of 2ps, all systems were equilibrated at 300 K for 2000ps. Using the isotropic position scaling approach and a pressure relaxation duration of 1ps, the pressure was held constant during the equilibration. Finally, a 110ns MD simulation was run for each system, adopting the equilibration protocol in periodic boundary conditions.

#### 2.16.1 Post dynamics assessment

The CPPTRAJ module of AMBER20 was used to analyze the output trajectory of vaccine-TLRs complexes. Using Cα atoms of each system, Root means square deviation (RMSD) and Root mean square fluctuation (RMSF) was computed as described elsewhere (67). In addition, the structural alteration during the simulation was quantified with the radius of gyration applying the equation (67). Further, the solvent-accessible surface area (SASA) was computed to study protein’s surface characteristics. In order to compute all hydrogen bonds between vaccine and receptor complex, the threshold distance and angel were retained at 3.5Å and 120° between the Hydrogen bond donor and acceptor atoms.

#### 2.16.2 Gibbs free energy distribution

The conformational free energy values of the complexes were studied at both the stable and transient stages. The CPPTRAJ package of AMBER 20 (67) was employed to investigate the systems’ Free Energy Landscape (FEL). Employing PC1 and PC2 principal components, the trajectories data were split into 100 bins. The most fluctuating values are PC1 and PC2. As a result, bins with no population were artificially restricted to a population size of 0.5 during the free energy calculations. Free energy was calculated and expressed in kcal/mol at 300°C.

### 2.17 Estimation of binding free energy

Binding free energies of the complexes were estimated through Molecular Mechanics/Generalized Born Surface Area (MM/GBSA) method (70) using the below equation (71):


(3) ΔGbind=ΔGR+L−(ΔGR+ΔGR)

Protein-ligand complex energy is ΔGR + L, apo protein energy is ΔGR, and ligand energy is ΔGL. Each free energy term(ΔG) in the above equation was computed as described (72).

## 3 Results

### 3.1 Protein sequence selection

Initially the structural and non-structural proteins sequences of NeoCoV were retrieved from NCBI to formulate a multi-epitope vaccine (**Table S1**). Structural proteins aid the virus in invading the host and synthesizing particles while non-structural proteins (NSPs) mediate the viral replications and structural protein synthesis. Followed by infection, these proteins trigger a distinct immunological response. Antigenicity, allergenicity, and transmembrane helices predictions were made on extracted sequences prior to their downstream analysis. All four structural proteins were found antigenic. Amongst NSPs, only ORF4a, ORF4b, ORF8b, ORF5b, viral protease, NSP10, ADP Binding module, and Coronavirus endopeptidase C30 were predicted as antigenic. With an antigenicity score of 0.61, structural protein ‘Nucleocapsid’ (N) was found as the most antigenic viral component of NeoCoV. The position and residue range of the viral protein sequences and vaccine constructs were determined using TMHMM Server. The selected sequence length of the envelope protein (39-50, 97-219), nucleocapsid protein (1-12), spike protein (1310-1344), and ORF4b (117-128, 171-284) were predicted inside the cell. All other selected components of the virus’ structural and NSPs were localized outside the cell (**Table 1**).

**Table 1:** NeoCoV proteins’ antigenicity prediction (With a viral model, threshold = 0.4). The TMHMM Server v2.0 was used to predict transmembrane helices in proteins.

Viral Component	Position	Aminoacids	VaxiJen Score	Probability	Localization
**E-Protein**	outside	1-19	0.4707	Non-allergen	Cell membrane
	TMhelix	20-38			
	inside	39-50			
	TMhelix	51-70			
	outside	71-73			
	TMhelix	74-96			
	inside	97-219			
**M-Protein**	outside	1-414	0.4879	Non-allergen	Endoplasmic reticulum, Membrane
**N-Protein**	inside	1-12	0.6193	Non-allergen	Cytoplasm, Soluble
	TMhelix	13-35			
	outside	36-82			
**S-Protein**	outside	1-1286	0.4988	Non-allergen	Cell membrane
	TMhelix	1287-1309			
	inside	1310-1344			
**ORF4a**	outside	1-103	0.4405	Non-allergen	Extracellular, Soluble
**ORF4b**	outside	1-109	0.5082	Non-allergen	Nucleus, Soluble
**ORF8b**	outside	1-199	0.4997	Non-allergen	Peroxisome, Membrane
**ORF5b**	outside	1-93	0.4567	Non-allergen	Endoplasmic reticulum, Membrane
	TMhelix	94-116			
	inside	117-128			
	TMhelix	129-146			
	outside	147-150			
	TMhelix	151-170			
	inside	171-284			
**Protease (PLPro)**	outside	1-313	0.4811	Non-allergen	Extracellular, Soluble
**NSP10**	outside	1-120	0.6372	Non-allergen	Extracellular, Soluble
**ADP Binding module**	outside	1-101	0.5731	Non-allergen	Mitochondrion, Soluble
**Coronavirus endopeptidase C30**	outside	1-278	0.575	Non-allergen	Extracellular, Soluble

### 3.2 Prediction and selection of linear B-cell epitopes

B-cell epitopes are antigen fragments that attach to antibodies or immunoglobulin, thereby cause B-cells to produce an immunological response (73). Primer for B-cell epitopes were predicted by BCPREDS for all antigenic protein components of NeoCoV, while BepiPred and ABCPred were employed to validate BepiPred’s predictions. If any of the other two servers did not predict a B-cell epitope predicted by the primary server, it was discarded. The predicted epitopes were preferred based on their non-allergenicity, antigenicity, non-toxicity, and surface accessibility. Epitopes that showed antigenicity score ≥0.4 were chosen for vaccine formulation. For all viral proteins, a total of 92 epitopes were predicted, however, only 19 B-cell epitopes fulfilled the criteria which were selected for further analysis. Despite showing antigenicity, no B-cell epitopes for ORFa, ORFb, and NSP10 were selected due to not fulfilling the set criteria for prioritizing the epitopes (**Table 2**).

**Table 2:** Selection of B-cell epitopes. Twenty amino acid-long B-cell epitopes were predicted with BCPREDS and validated with BepiPred and ABCPred servers.

Viral Component	Pos.	Epitopes	B-score	AT-score	A-score	Allergenicity	Toxicity
E	61	*TG* ***RS*** *VYVKFQ* ***ESKPPLPP*** *E*E	0.99	0.54	0.83	NA	NT
M	1	**MSNM*TQLSEQQIIAIIKDWN***	0.89	0.50	0.82	NA	NT
N	192	***GNSSRGASPGPSGVGAPG*GD**	1	0.54	0.85	NA	NT
	20	**TNQPRG*RGRNPKPRAAPNTT***	1	0.50	0.89	NA	NT
	361	**PKKEKK*QKAPKEESNDQEMA***	1	0.41	0.82	NA	NT
	124	*E* ***EGATDAPSTFGTRN*PNNDS**	0.99	0.57	0.74	NA	NT
S	1207	***NNLPPPLLSNSTGTD*FKDEL**	0.99	0.67	0.82	NA	NT
	17	*ANAKI* ***VTLPGNDATGY*CPSV**	0.99	0.53	0.8	NA	NT
	532	***NSPTT*** *GQLWAYN* ***F*GGVPYRV**	0.97	0.53	0.82	NA	NT
	84	***DLG*** *TQYVYSASN* ***HK*STANDA**	0.97	0.56	0.73	NA	NT
	506	***NYGATNKDDVV*KPGGRASQQ**	1	0.46	0.88	NA	NT
	189	***LLQPRTESKCPGNSN*** YVSYF	0.96	0.80	0.73	NA	NT
	842	*ESV* ***KTPQTVPLTTGFGG*** *EF*N	0.73	0.47	0.69	NA	NT
ORF8b	59	***LGIGGDRTERLTQEMELSN*W**	0.99	0.98	0.67	NA	NT
ORF5b	9	***KPVQLVPVSPVDHGG*ESNDS**	0.99	0.90	0.91	NA	NT
PLPro	286	*KF* ***DSGTLSKASD*** *W*KCKVTDV	0.83	0.93	0.69	NA	NT
	32	FFNGA**DI*SDTIPDEKQHGCS***	0.8	0.45	0.9	NA	NT
ADP Binding Module	29	*GAVQQ* ***ESDEYILTRGPLQVG***	0.93	0.45	0.76	NA	NT
Coronavirus endopeptidase C30	30	*LVSMTNHSFS* ***VQKHVGA*P**AN	0.87	0.57	0.79	NA	NT

Full length epitope is the prediction of BCPREDS. Bold letters show the BepiPred predicted epitope and letters written in italics are the ABCPred predicted epitope. Pos, Position; B-score, BCPred predicted score; AT-score, VexiJen antigenicity score; A-score, ABCpred predicted score; NT, Non-toxic; NA, Non-allergen. The projected epitope with an antigenicity score of ≥ 0.4 was considered antigenic.

### 3.3 Prediction and selection of potential MHC class-I binding epitopes

The final list of identified epitopes for each viral antigenic component are given in **Table 3**. Briefly, 69 NeoCoV epitopes were predicted which showed significant binding interactions with a minimum of four HLA I subtypes. Prior to selection for further investigation, T cell epitopes were rated based on strong IEDB score, binding affinity (≥ 4 HLA1 subtypes), B-cell epitope overlap, considerable antigenicity, non-allergenicity, and non-toxicity. Following these criteria, eight epitopes for S-protein, four for M-protein, two for N-protein and ORF4b, and a single epitope for each E-protein and Coronavirus endopeptidase C30 were retained for vaccine design. The overall HLA score was computed for each epitope. Interestingly, these epitopes had never been investigated experimentally before, indicating that they are novel predictions. Upon applying the IEDB SMM-align method, most of the shortlisted epitopes showed high binding affinity (IC_50_ value >500nM) with their respective HLA supertype allele. The CTLPred server also confirmed these selected epitopes as potent Cytotoxic T-lymphocyte (CTL) epitopes following the combined approach as well as ANN and SVM thresholds of 0.51 and 0.36, respectively (**Table S2**).

**Table 3:** List of prioritized MHC-I (CTL) binding epitopes by NetCTL 1.2 server.

Protein	MHC-I Epitopes	No. of HLASuper-types	HLA supertypes (IC_50_ nM)	Total HLAscore	AT	Allergenicity	Toxicity	Immunogenicity
E	FTVVCAITL	4	A2 (380.29), B8 (135), B39 (245.13), B62 (95.11)	3.628	1.05	NA	NT	0.17427
M	GTNSGVAIY	4	A1 (77.11), A3 (259), A26 (133), B62 (142.97)	5.428	0.46	NA	NT	0.01407
	ALSIFSAVY	4	A1 (282), A3 (68.46), B58 (101.2), B62 (170.70)	4.529	0. 61	NA	NT	0.09563
	YPSRSMTVY	5	A1 (246.49), A26 (273.6), B7 (529.53), B8, B62 (66.87)	4.734	0. 92	NA	NT	0.27114
	LLITIVLQY	5	A1 (126.26), A3 (580.50), A26 (111.73), B58 (70.81), B62 (567.85)	5.823	0.87	NA	NT	0.17368
N	STPAQNAGY	5	A1 (122.99), A3 (874.13), A26 (187.76), B58 (811.5), B62 (98.00)	7.243	0.44	NA	NT	0.0303
	SAFMGMSQF	5	A26 (189.00), B7 (351.4), B8 (669.1), B58 (408.43), B62 (27.24)	3.793	0.44	NA	NT	0.4783
S	WSYTGSSFY	5	A1 (207), A3 (405.32), A26 (128.17), B58 (415.24), B62 (86.49)	6.884	1.06	NA	NT	0.15609
	YSTNITHLL	5	A1 (96.5), A2 (97.43), B39 (700.486), B58 (351.8), B62 (479.036)	4.884	0.62	NA	NT	0.19305
	ISYAGAYSY	4	A1 (766.56), A3 (404.38), B58 (40.114), B62 (59.841)	5.841	0.80	NA	NT	0.00822
	SVTIADPGY	4	A1 (112.5), A26 (436.77), B58 (385.76), B62 (112.78)	3.538	1.07	NA	NT	0.01912
	ALQEVVKAL	4	A2 (195.934), B7 (299.5), B8 (307.99), B62 (401.678)	3.634	0.52	NA	NT	0.01693
	TMKKIYPAL	5	A2 (110.358), A24 (401.48), B8 (119), B39 (103.48), B62 (615.176)	4.943	0.52	NA	NT	0.14738
	MVYVITVKY	4	A3 (143.81), A26 (636.223), B58 (311.386), B62 (402.71)	4.385	0.91	NA	NT	0.11532
	FLFATVPIY	5	A3 (509.09), A26 (488.03), B39 (266.701), B58 (266.701), B62 (46.45)	6.127	0.56	NA	NT	0.22243
ORF4b	HSPGKNLRY	5	A1 (491.53), A3 (430.28), A26 (220.95), B58 (274.79), B62 (410.42)	5.421	0.43	NA	NT	0.16471
	SVVTQPTHY	4	A1 (532.9), A3 (344.9), A26 (126), B62 (128.60)	5.572	0.53	NA	NT	0.01912
Coronavirus endopeptidase C30	ASFSVLACY	5	A1 (114.33), A3 (91.58), A26 (339.2), B58 (300.82), B62 (127.94)	5.841	0.62	NA	NT	0.09719

AT, Antigenicity score (HLA supertype with binding affinity scores ≥ 0.75 were taken); NA, Non-allergen; NT, Non-toxic.

### 3.4 Prediction and selection of potential MHC class-II binding epitopes

Using NetMHCIIpan version 3.2, a total of 66 putative MHC-II epitopes (15-mer) in the antigenic NeoCoV proteins were predicted to bind with at least three specific HLA DRB alleles (39). Before further analysis, epitopes were evaluated for strong binding affinity (≥ 3 HLA II subtypes), B-cell epitope overlap, considerably antigenicity, non-allergenicity, and non-toxicity. Following these specifications, seven epitopes for S-protein, four epitopes for N-protein, two each for N-protein, PLPro, NSP10, and ADP Binding module, and single epitopes for each ORF4a, ORF4b, and ORF5b were retained for vaccine design (**Table 4**). These epitopes showed IC_50_ >500 nM with their respective HLA II alleles using IEDB SMM-align method. Furthermore, these epitopes had never been investigated experimentally which confirms their predictions. The ability of the selected epitopes to stimulate IFN-γ secretion through helper T lymphocytes (HTLs or MHC-II epitopes) was determined by the IFNepitope server. The majority of shortlisted HTL epitopes were also predicted as IL4 and IL10 inducers (**Table S3**), thus, these epitopes can be classified as putative HLA II T-cell epitopes capable of activating CD4+ T-cells. Nine-mer epitopes of the final 15-mer peptides possess IC_50_ values >50nM with the DRB1*0101 allele (**Table S4**).

**Table 4:** List of prioritized MHC-II (HTL) binding epitopes by NetMHCIIpan.

Protein	MHCII Epitopes	No. of HLA alleles	HLA alleles/IC50 value (nM)	AT	Allerg.	Tox.
M	LPNEITVAKPNVLIA	3	DRB1*0101/(10.96), DRB1*0701/(27.53), DRB1*1501/(111.34)	0.41	NA	NT
	LIALKMVKRQSYGTN	3	DRB1*0301/(180.49), DRB1*1101/(46.15), DRB1*1301/(17.3)	1.00	NA	NT
N	TKSFNMVQAFGLRGA	3	DRB1*0101/(3.56), DRB1*0701/(7.18), DRB1*1501/(31.54)	0.82	NA	NT
	PKVITKKDAAAAKNK	6	DRB1*0101/(213.03), DRB1*0401/(116.37), DRB1*0801/(105.17), DRB1*1101/(77.54), DRB1*1301/(212.59), DRB1*1501/(115.52)	0.75	NA	NT
	SGAIKLDPKNPNYNK	4	DRB1*0301/(96.66), DRB1*0801/(177.66), DRB1*1101/(69.44), DRB1*1301/(79.35)	1.11	NA	NT
	PRWYFYYTGTGPEAA	4	DRB1*1501/(356.18), DRB1*0401/(155.66), DRB1*0701/(90.79), DRB1*0801/(233.88)	0.76	NA	NT
S	STSYYSAKPVGAYYE	5	DRB1*0101/(6.26), DRB1*0401/(52.57), DRB1*0701/(88.17), DRB1*0801/(274.17), DRB1*1101/(96.90)	0.43	NA	NT
	PEPITTLNTRYVAPQ	5	DRB1*0101/(36.29), DRB1*0401/(514.57), DRB1*0701/(422.39), DRB1*1301/(126.66), DRB1*1501/(119.58)	0.82	NA	NT
	ISYDIYGITGTGVFQ	3	DRB1*0101/(19.77), DRB1*0701/(56.03), DRB1*1501/(155.33)	0.67	NA	NT
	YVAGYKVLPPLMDVN	3	DRB1*0101/(18.05), DRB1*0401/(81.41), DRB1*1101/(88.74)	0.48	NA	NT
	GTQYVYSASNHKSTA	4	DRB1*0101/(34.89), DRB1*0401/(96.98), DRB1*0701/(153.93), DRB1*1101/(156.61)	0.45	NA	NT
	TQYVYSASNHKSTAN	3	DRB1*0101/(34.76), DRB1*0401/(96.43), DRB1*1501/(225.61)	0.42	NA	NT
	IIGFHSDDGNYYCVA	3	DRB1*0301/(112.99), DRB1*0401/(258.69), DRB1*1101/(493.8)	0.46	NA	NT
ORF4a	TAKYTPAPGTSLHPV	3	DRB1*0101/(17.07), DRB1*0401/(109.35), DRB1*0701/(448.9)	0.71	NA	NT
ORF4b	ARDISPIAVFLRNVR	3	DRB1*0301/(28.77), DRB1*1301/(368.61), DRB1*1501/(91.19)	1.04	NA	NT
ORF5b	STVFVPATRDSVPLH	3	DRB1*0301/(436.22), DRB1*0801/(334.17), DRB1*1101/(41.04)	0.59	NA	NT
PLPro	SPDFVAFNVFHGMET	6	DRB1*0101/(42.9), DRB1*0401/(84.87), DRB1*0701/(137.13), DRB1*0801/(194.89), DRB1*1101/(173.16), DRB1*1501/(52.95)	0.71	NA	NT
	FRTVVLNNKNSYRSQ	3	DRB1*0301/(65.99), DRB1*1101/(180.05), DRB1*1501/(21.08)	0.83	NA	NT
NSP10	KGKFVQIPSQCTRDP	5	DRB1*0101/(7.91), DRB1*0401/(34.54), DRB1*070I/(61.37), DRB1*0801/(191.62), DRB1*1101/(88.24)	0.57	NA	NT
	GTGIAISVKPESTAD	4	DRB1*0301/(102.9), DRB1*0801/(75.29), DRB1*1101/(350.6), DRB1*1301/(350.6),	1.21	NA	NT
ADP Binding module	SKCYRAMNAYPLVVT	3	DRB1*0101/(2.95), DRB1*0701/(12.61), DRB1*1501/(30.76)	0.70	NA	NT
	AKNILHVVGPDARAK	3	DRB1*0101/(12.5), DRB1*0301/(897.1), DRB1*1501/(65.47)	0.66	NA	NT
CoV endopeptidase C30	QQLYTGFQGKQILGS	3	DRB1*0101/(51.5), DRB1*0701/(118.1), DRB1*1501/(220.03)	0.41	NA	NT
	TGTFTVIMRPNYTIK	3	DRB1*0801/(72.77), DRB1*1101/(22.62), DRB1*1301/(25.63)	0.77	NA	NT

Predicted IC_50_ value by the IEDB tool (SMM method) is shown in parenthesis for each epitope with the respective HLA allele. AT, Antigenicity score; Allerg, Allergenicity; Tox, Toxicity (HLA allele with binding affinity scores≥ 0.75 were taken); NA, Non-antigenic; NT, Non-toxic. * is a part of nomenclatures indicating the method is molecular typing.

### 3.5 Population coverage analysis

**Figure 2:**
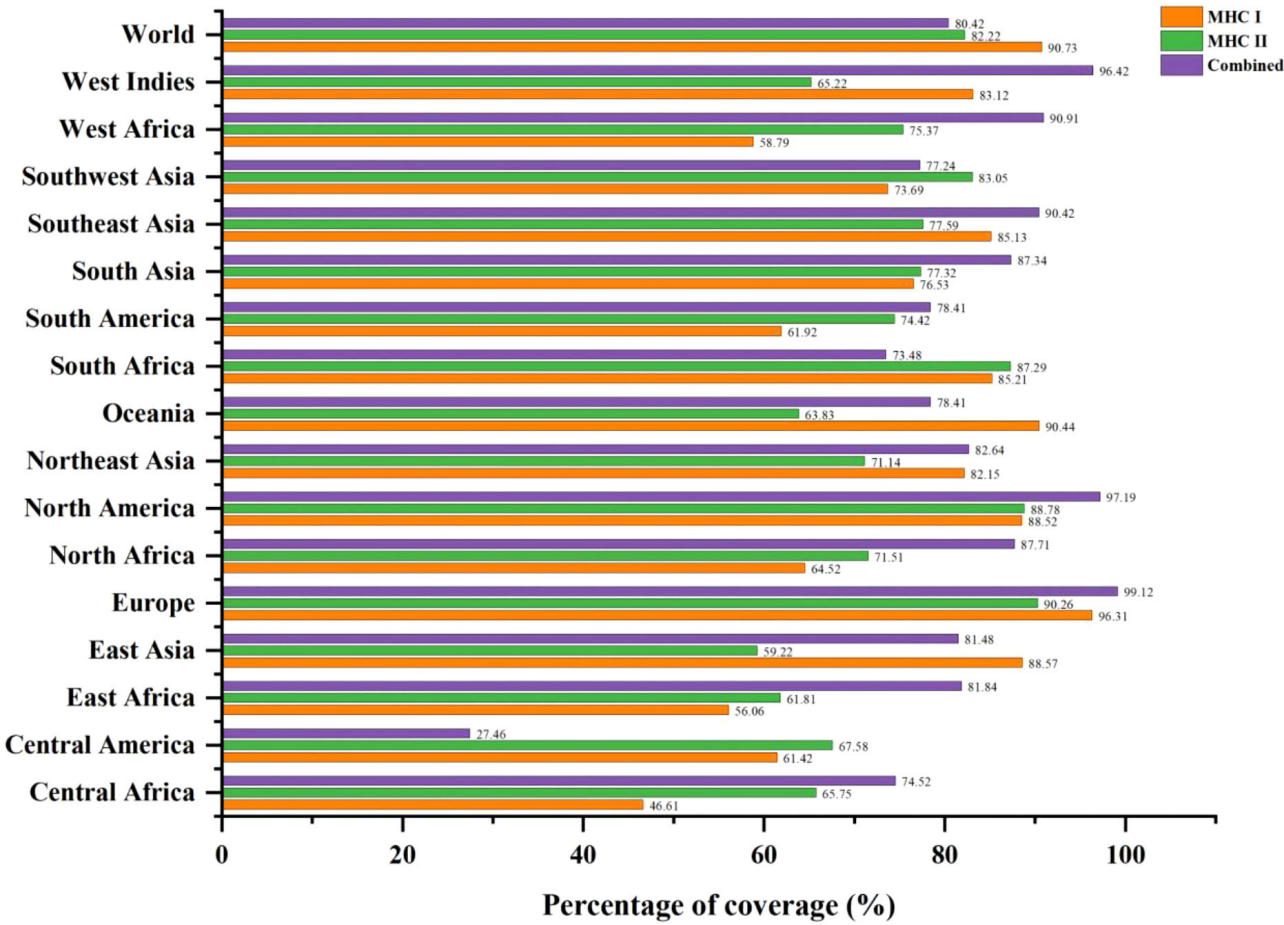
Global population coverage of selected T-cell epitopes (MHC-I, MHC-II, and combined MHC) based on their corresponding HLA binding alleles.

The MHC-I and MHC-II epitopes have 90.73% and 82.22% worldwide coverage, respectively, according to the population coverage study. We also focused on their combined population coverage since a vaccine construct contains both types of MHC epitopes. The total combined coverage accounted for 80.42% of the world’s population. For combined MHC class-I and II epitopes, Europe has highest population coverage (99.12%), followed by North America (97.19%), West Indies (96.42%), West Africa (90.91%), Southeast Asia (90.42%), North Africa (87.71%), South Asia (87.34%), Northeast Asia (82.64%), East Africa (81.84%), East Asia (81.48%), South America and Oceania (78.41% each), Southwest Asia (77.24%), South Africa (73.48%), Central Africa (74.52%), and Central America (27.46%). The relative population coverages of individual MHC classes and combined MHC epitopes are shown in **Figure 2**.

### 3.6 Physiochemical profile of epitopes

Using the Expasy ProtParam server, the physicochemical profile of final peptides was evaluated (**Tables S3, S5** and **S6**). The instability index scores of 12 MHC class I and 18 MHC class II peptides predicted them as stable in the test tube (>40). Similarly, the high aliphatic index value showed that 13 MHC-I and 20 MHC-II final epitopes are thermostable. Likewise, the negative GRAVY score shows that most MHC-I epitopes and all MHC-II epitopes are hydrophilic. The projected half-life in mammalian cells for CTL epitopes ranged from 1.1 hr to 30 hrs, whereas for HTL epitopes, it was 1.1h to >20hrs. Most of the final CTL and HTL peptides were computed as acidic (isoelectric point< 7), and respectively their molecular weight ranged from 880.95–1115.40 Da and 1445.5–1826.05 Da. Furthermore, 13/19 B-cell epitopes were stable, 13 were predicted thermostable, and the majority indicated the possibility of interaction with water. The estimated half-life in mammalian cells ranged from 1h-30hrs, and most were projected as acidic with molecular weight varying from 1683.71 to 2363.73 Da.

### 3.7 Vaccine construction

To stimulate an immune response against NeoCoV, four vaccines were developed leveraging the prioritized epitopes. Three adjuvants, β-defensin, 50S ribosomal protein L7/L12, and HBHA protein, were used to create four distinct vaccines (Neo-1–4). The PADRE sequence is a pan HLA-DR epitope peptide used to improve vaccination efficacy while minimizing toxicity. As a potential immunogen, the PADRE sequence enables the binding of CTL epitopes with various class II MHC molecules with high affinity (44). During the design phase, the selected epitopes were also conjugated with the appropriate linkers. The final vaccine length of Neo-1–4 is 358, 438, 448, and 415 bp, respectively. The sequence and length of the four vaccine constructs are presented in (**Table 5**).

**Table 5:** The predicted vaccine constructs for NeoCoV.

Vaccine Name	Adjuvant	Epitope count	Length	Vaccine Constructs
NeoCoV vaccine- 1 (Neo-1)	β-defensin	HTL (6) CTL (5) B-cell (5)	358	GIINTLQKYYCRVRCAVLSCLPKEEQIGKCSTRGRKCCRRKKEAAAK**AKFVAAWTLKAAA**GGGSVFTVVCAITLAAYGTNSGVAIYAAYSTPAQNAGYAAYWSYTGSSFYAAYHSPGKNLRYGGGSLPNEITVAKPNVLIAGPGPGTKSFNMVQAFGLRGAGPGPGSTSYYSAKPVGAYYEGPGPGTAKYTPAPGTSLHPVGGPGSTVFVPATRDSVPLHGPGPGSPDFVAFNVFHGMETGGGS*TGRSVYVKFQESKPPLPPEE*KK*MSNMTQLSEQQIIAIIKDWN*KK*GNSSRGASPGPSGVGAPGGD*KK*NNLPPPLLSNSTGTDFKDEL*KK*LGIGGDRTERLTQEMELSNW*HHHHHH
NeoCoV vaccine- 2 (Neo-2)	Ribosomal protein	HTL (6) CTL (5) B-cell (5)	438	MAKLSTDELLKEMTLLELSDFVKKFEETFEVTAAAPVAVAAAGAAPAGAAVEAAEEQSEFDVILEAAGDKKIGVIKVVREIVSGLGLKEAKDLVDGAPKPLLEKVAKEAADEAKAKLEAAGATVTVKEAAAK**AKFVAAWTLKAAA**GGGSASFSVLACYAAYALSIFSAVY**AAY**SAFMGMSQFAAYSAFMGMSQFAAYYSTNITHLLGGGSKGKFVQIPSQCTRDPGPGPGSKCYRAMNAYPLVVTQQLYTGFQGKQILGSGPGPGLIALKMVKRQSYGTNGPGPGPKVITKKDAAAAKNKGPGPGPEPITTLNTRYVAPQGGGS*KPVQLVPVSPVDHGGESNDS*KK*KFDSGTLSKASDWKCKVTDV*KK*GAVQQESDEYILTRGPLQVG*KK*LVSMTNHSFSVQKHVGAPAN*KK*TNQPRGRGRNPKPRAAPNTT*HHHHHH
NeoCoV vaccine- 3 (Neo-3)	Heparin-binding hemagglutinin	HTL (6) CTL (3) B-cell (5)	448	MAENPNIDDLPLAALGAADLALATVNDLIANLRERAEETRAETRTRVEERRARLTKFQEDLPEQFIELRDKFTTEELRKAAEGYLEAATNRYNELVERGEAALQRLRSQTAFEDASARAEGYVDQAVELTQEALGTVASQTRAVGERAAKLVGIELEAAAK**AKFVAAWTLKAAA**GGGSLLITIVLQYAAYISYAGAYSYAAYSVVTQPTHYGGGSARDISPIAVFLRNVRGPGPGFRTVVLNNKNSYRSQGPGPGGTGIAISVKPESTADGPGPGAKNILHVVGPDARAKGPGPGTGTFTVIMRPNYTIKGPGPGSGAIKLDPKNPNYNKGGGS*PKKEKKQKAPKEESNDQEMA*KK*ANAKIVTLPGNDATGYCPSV*KK*FFNGADISDTIPDEKQHGCS*KK*EEGATDAPSTFGTRNPNNDS*KK*NSPTTGQLWAYNFGGVPYRV*HHHHHH
NeoCoV vaccine- 4 (Neo-4)	Ribosomal protein	HTL (6) CTL (5) B-cell (4)	415	MSDINKLAENLKIVEVNDLAKILKEKYGLDPSANLAIPSLPKAEILDKSKEKTSFDLILKGAGSAKLTVVKRIKDLIGLGLKESKDLVDNVPKHLKKGLSKEEAESLKKQLEEVGAEVELKEAAAK**AKFVAAWTLKAAA**GGGSSVTIADPGYAAYALQEVVKALAAYTMKKIYPALAAYMVYVITVKYAAYFLFATVPIYGGGSPRWYFYYTGTGPEAAGPGPGISYDIYGITGTGVFQGPGPGYVAGYKVLPPLMDVNGPGPGGTQYVYSASNHKSTAGPGPGTQYVYSASNHKSTANGPGPGIIGFHSDDGNYYCVAGGGS*DLGTQYVYSASNHKSTANDA*KK*NYGATNKDDVVKPGGRASQQ*KK*LLQPRTESKCPGNSNYVSYF*KK*ESVKTPQTVPLTTGFGGEFN*HHHHHH

The sequences highlighted in bold are the pan HLA DR-binding epitope (PADRE), whereas the italics sequences are the B-cell epitopes.

### 3.8 Assessment of antigenicity, allergenicity, toxicity, and surface accessibility

Besides various adjuvants employed in the design, all vaccine constructs were tested for antigenic property, safety, allergenicity, and surface accessibility (**Table 6**). The selected sequence length of Neo-1 (1-49), Neo-2 (1-150), and Neo-4 (1-177) were predicted inside the cell. All other selected components of these constructed vaccines as well as the complete sequence length of Neo-3, were localized outside the cell. The antigenicity score of the final vaccine constructs ranged from 0.44–0.56 and 0.92–0.97 according to the VexiJen and ALLERGENPro predictions, respectively, indicating that our final vaccine constructs have high antigenicity. The final vaccine constructs and its components were predicted to be non-allergenic by the AllergenFP 1.0 and the AllerTOP 2.0 server. Moreover, the ToxinPred server revealed our constructed vaccines and every subunit as non-toxic.

**Table 6:** Allergenicity, antigenicity, toxicity, surface accessibility, and physicochemical characteristics of proposed polypeptide-based vaccine constructs (Neo-1-4).

Properties	Neo-1	Neo-2	Neo-3	Neo-4
Sol-Pro	0.946675	0.993184	0.869426	0.757577
Protein Sol	0.517 (soluble)	0.641	0.517	0.515
Molecular weight	37678.63 Da (avg.)	45770.41 Da	47868.55 Da	44042.97 Da
Formula	C_1676_H_2596_N_472_O_496_S_12_	C_2038_H_3250_N_562_O_608_S_13_	C_2099_H_3329_N_615_O_658_S_5_	C_1991_H_3095_N_521_O_595_S_6_
Theoretical pI	9.56(basic)	9.47 (basic)	8.97 (basic)	9.15 (basic)
Ext. coefficient	47,705 M^-1^ cm^-1^	30,620 M^-1^ cm^-1^	34, 965 M^-1^ cm^-1^	52,845 M^-1^ cm^-1^
Instability index	35.94 (stable)	29.00 (stable)	33.82 (stable)	25.29 (stable)
Aliphatic index	60.67 (thermostable)	73.63 (thermostable)	69.62 (thermostable)	75.98(thermostable)
Grand average of hydropathicity (GRAVY)	-0.421(hydrophilic)	-0.245 (hydrophilic)	-0.586 (hydrophilic)	-0.347 (hydrophilic)
Half-Life (satisfactory)	30 hrs (Mammalian reticulocytes, *in vitro*). >20 hrs (yeast, *in vivo* >10 hrs (*Escherichia coli*, *in vivo*).	30 hrs (Mammalian reticulocytes, *in vitro*). >20 hrs (yeast, *in vivo*). >10 hrs (*Escherichia coli*, *in vivo*).	30 hrs (Mammalian reticulocytes, *in vitro*). >20 hrs (yeast, *in vivo*). >10 hrs (*Escherichia coli*, *in vivo*).	30 hrs (Mammalian reticulocytes, *in vitro*). >20 hrs (yeast, *in vivo*). >10 hrs (*Escherichia coli*, *in vivo*).
Allergenicity	AllerTop (Non-allergen), Allergen FP (Non-allergen)	AllerTop (Non-allergen), Allergen FP (Non-allergen)	AllerTop (Non-allergen), Allergen FP (Non-allergen)	AllerTop (Non-allergen), Allergen FP (Non-allergen)
Antigenicity	ANTIGENpro (0.97), VexiJen (0.56)	ANTIGENpro (0.95), VexiJen (0.52)	ANTIGENpro (0.93), VexiJen (0.53)	ANTIGENpro (0.92), VexiJen (0.44)
Toxicity	Non-toxic	Non-toxic	Non-toxic	Non-toxic
Surface accessibility	Position: residues Inside: 1-49 TMhelix: 50-72 Outside: 73-358	Position: residues Inside: 1-150 TMhelix: 151-173 Outside: 174-478	Outside	Position: residues Inside: 1-177 TMhelix: 178-200 Outside: 201-415

### 3.9 Vaccine physiochemical properties evaluation

The numerous vaccine components and their individual properties are listed in **Table 6**. The projected values indicate that our final vaccines and each subunit are well soluble. The molecular weight of four vaccine constructs [Neo-1 (37678.63 Da), Neo-2 (45770.41 Da), Neo-3 (47868.55 Da), Neo-4 (44042.97 Da)] is less than 110 kDa, which is regarded as suitable for vaccine development (74). The theoretical isoelectric point (pI) of four vaccines construct ranged from 8.57 to 9.56, which falls within the normal pH range. The instability index of Neo-1–4 was ≤40; thus, we can expect our vaccine protein is stable. Our vaccines Neo-1–4 have an aliphatic index of 60.67, 73.63, 69.62, and 75.98, respectively, which is a significant indicator of thermostability at different temperatures. The grand average of hydropathicity (GRAVY) for the four constructed vaccines was negative, indicating the vaccines are hydrophilic in nature (75). The half-life of all the designed vaccines was determined in mammalian immature red blood cells (30 hours, *in vitro*), in yeast (>20 hours, *in vivo*), and *Escherichia coli* (>10 hours, *in vivo*). The instability index of Neo-1-4 (>40) suggest their stability in test tube. Additionally, BLASTp against the human genome revealed that the sequences had no human counterpart. Proteins that are human homologs may induce autoimmunity unexpectedly due to off-target reactions with the host proteins (76). Overall, the physicochemical examination of the polypeptide-based constructs reveals that these are appropriate for immunological applications.

### 3.10 Human homology and novelty analysis

The selected B-cell, CTL, and HTL epitopes and the final vaccine constructs, were subjected to BLASTp homology search against human proteome (taxid: 9606). The query coverage of shortlisted epitopes and vaccine constructs did not significantly resemble human protein sequences (Neo-1 = 7%, Neo-2 = 21%, Neo-3 = 21%, Neo-4 = 14%). Thus, the predicted vaccines would not elicit autoimmune reactions in the host. Besides, all prioritized B-cell, MHC-I, and MHC-II epitopes were indicated as novel prediction upon checking the IEDB database.

### 3.11 Prediction of secondary structure

**Figure 3:**
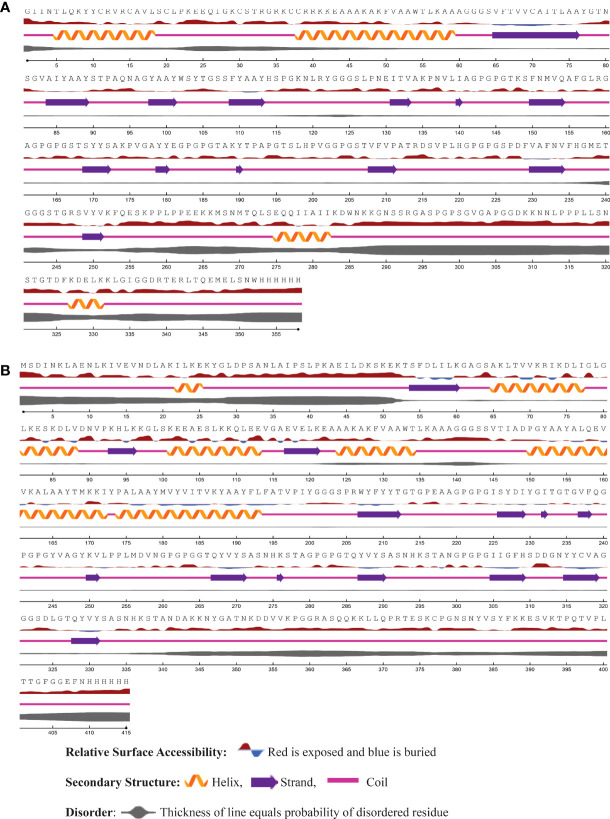
The predicted secondary structure features, relative surface accessibility, and disorder regions of **(A)** Neo-1 **(B)** Neo-4 using NetsrurfP-3.0 server.

Using the NetSurfP - 3.0 server, we predicted the secondary structure of our final vaccines. According to the projected secondary structure, the Neo-1 vaccine have 15.59% α-helix, 13.4% β-strand, and 70.75% coil. The Neo-2 and Neo-3 vaccine showed 9.13% and 10.26% α-helix, 22.6% and 18.97% β-strand, 68.27% and 70.75% secondary coil structure, respectively. The secondary structure of the Neo-4 comprises of 15.42% α-helix, 35% β-strand, and 62.65% coil. In addition, solvent accessibility (ACC) and disorder areas (DISO) of Neo-1-4 were also estimated. Neo-1 has 358 residues, 94% of which are projected to be exposed and only 6% are buried, while 60 residues (16.71%) are expected to be in disordered regions. Neo-2 vaccine construct is predicted to have 83.10% of residues exposed and 16.89% hidden, whereas 88 residues (20%) of Neo-2 are expected to lie in disordered regions. Similarly, in Neo-3, 89.5% of residues are projected to be exposed and 10.5% to be buried, and 47 residues (10.49%) are anticipated to be in disordered regions. Likewise, 83.17% residues of Neo-4 are exposed and 16.89% hidden, while 80 residues (17.70%) are expected to locate in disordered regions. The predicted secondary structure features, relative surface accessibility, and disorder regions of Neo-1–4 is depicted in **Figure 3** and **Figure S1**.

### 3.12 Tertiary structure modeling and refinement of constructed vaccine

**Figure 4:**
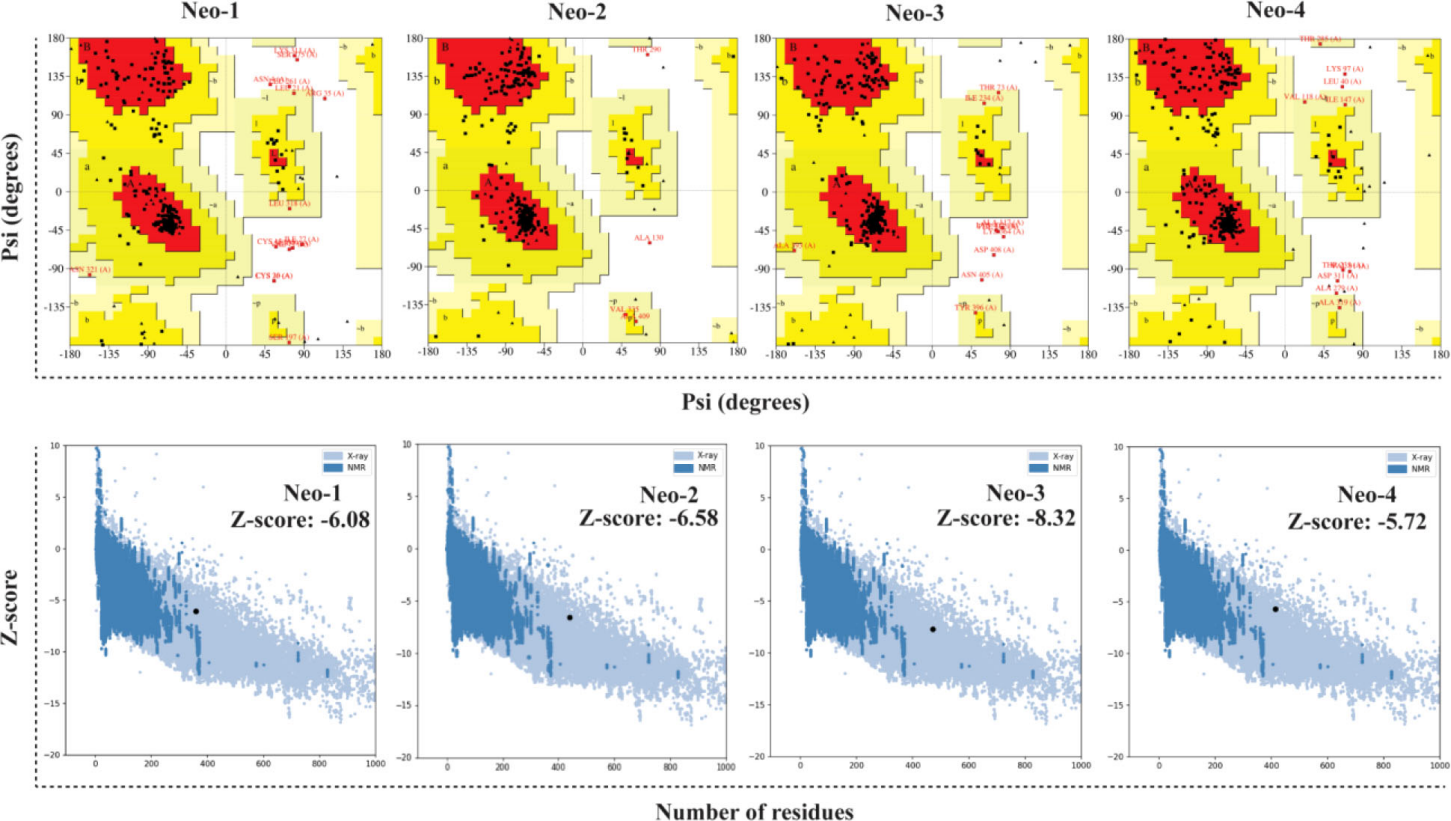
Validation of the predicted 3D structure models of NeoCoV vaccines with PROCHECK and ProSA-webserver.

The RoseTTAFold tool and GalaxyRefine server were employed to predict and improve the modeled structures of the final vaccine constructs. We picked model 1 for Neo-1, 2, and 4 and model 4 for Neo-3 based on the model quality of the five refined models projected by GalaxyRefine (**Table S7**). The ERRAT quality score, ProSA Z-score, and Ramachandran plot analysis were used to verify the overall model quality of the refined vaccine structures. ERRAT gave an overall quality score of 81.26, 95.87, 97.18, and 90.41 for Neo-1 to 4, respectively. The ProSA predicted Z-score of Neo-1 to 4 were -6.08, -6.58, -8.32, and -5.72, respectively, which is within the scoring range of similar-sized native proteins, suggesting high overall model quality. Using ProSA, we further checked the quality of the local model, and the residue scores are presented in **Figure 4**. The negative values indicate that the model structure is free of errors. We further used the PROCHECK server (77) for Ramachandran plot analysis. The refined model structure of Neo-1contains 81.6%, 13%, and 1.4% of residues in most favored, additionally allowed, and generously allowed regions, respectively, whereas only 4% of residues lie in disallowed regions. Neo-2 refined model has collectively 99.4% residues in allowed regions, while only 0.6% of residues lie in the disallowed region. Similarly, Neo-3 and Neo-4 refined modelled structures has 98.5% and 99.2% of residues in allowed regions and 1.5% and 0.8% in disallowed regions, respectively (**Figure 4** and **Table S8**). The final 3D-model of each vaccine construct is illustrated in **Figure S2**.

### 3.13 Prediction of conformational B-cell epitopes

The new protein’s structure and folding can lead to new conformational B-cell epitopes, prompting further predictions. Such epitopes in the refined modeled structure were predicted through the ElliPro server (34). **Tables S9, S10** enlists predicted discontinuous B-cell epitopes for all four vaccine constructs. Briefly, seven conformational B-cell epitopes were predicted for Neo-1 involving a total of 183 residues with a score ranging from 0.562 to 0.796. For Neo-2, eight conformational B-cell epitopes were predicted, which involved 212 residues with score in range of 0.526 to 0.981. Similarly, predicted conformational B-cell epitopes for Neo-3 (score ranged from 0.575 to 0.817) and Neo-4 (score ranged from 0.512 to 0.769) involved 219 and 211 residues, respectively.

### 3.14 Molecular docking analysis

The interactions between the antigenic molecule and immune receptor molecule at the molecular level are critical to successfully activating the transport of antigenic molecule and immune response (78, 79). To study possible interactions and binding energy, docking was performed between several immune receptors (TLR2, TLR3, TLR4, MHC-I and II) and developed vaccines (Neo-1–4). TLRs can elicit an immunological response upon viral recognition. We selected the best-docked complex among 30 docked poses based on the lowest energy score, indicating that the constructed vaccines fit in receptor efficiently with strong binding affinity. Amongst all TLRs, TLR3 showed the best binding with Neo-4 and Neo-1 with binding energies of -114.47 kcal/mol and -101.08 kcal/mol, respectively, followed by Neo-2 (-91.88 kcal/mol) and Neo-3 (-81.01 kcal/mol). For all these TLRs, Neo-1 and Neo-4 exhibited lower binding energies than Neo-2 and Neo-3. Furthermore, Neo-1–4 displayed good interaction energy (ranging from-76.52 to-85.43 kcal/mol) with both classes of MHC molecules. According to the docking study, the proposed vaccines and selected immune receptors have significant interactions and successful binding. The docking calculations are given in **Table 7**.

**Table 7:** Molecular docking results of Neo-1-4 with selected TLRs.

Vaccine construct	Receptor	Name of target	Score (kcal/mol)	E-Conf	E-Place
**Neo-1**	2Z7X	TLR2	-86.52	-7275.00	-19.84
	2A0Z	TLR3	**-101.08**	**-7271.54**	**-25.89**
	3FXI	TLR4	-82.04	-6219.70	-20.79
	1AKJ	MHC-I	-85.43	-7157.54	-20.21
	3L6F	MHC-II	-83.97	-6521.34	-19.54
**Neo-2**	3FXI	TLR4	-70.64	-10124.58	-32.51
	2A0Z	TLR3	-91.88	-10116.98	-26.57
	2Z7X	TLR2	-78.13	-8714.57	-31.01
	1AKJ	MHC-I	-81.21	-9324.76	-23.56
	3L6F	MHC-II	-78.54	-8731.90	-28.54
**Neo-3**	3FXI	TLR4	-68.93	-14795.16	-36.42
	2A0Z	TLR3	-81.01	-14808.04	-29.46
	2Z7X	TLR2	-86.32	-13277.29	-32.06
	1AKJ	MHC-I	-76.52	-13789.54	-32.65
	3L6F	MHC-II	-83.90	-14567.65	-34.67
**Neo-4**	3FXI	TLR4	-81.73	-8482.36	-30.68
	2A0Z	TLR3	**-114.47**	**-8515.66**	**-35.05**
	2Z7X	TLR2	-79.56	-7223.11	-33.44
	1AKJ	MHC-I	-84.76	-8675.12	-32.67
	3L6F	MHC-II	-81.87	-8234.65	-31.89

E-Conf, Energy of Confirmation; E-Place, Energy of Placement.

**Figure 5:**
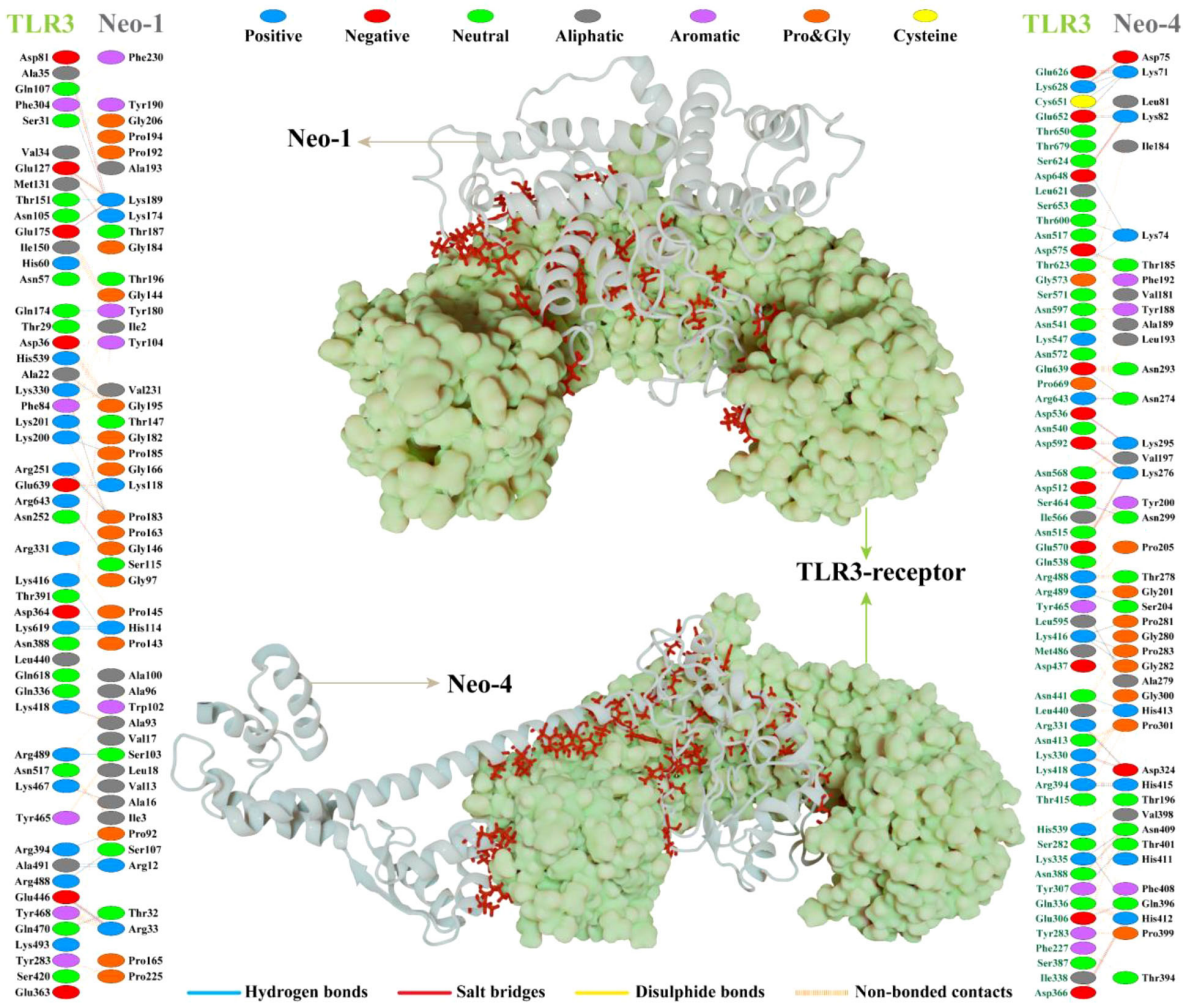
NeoCoV vaccine constructs (Neo-1 and Neo-4) docked with human Toll-like receptor-3 (TLR3). The best-docked vaccine-TLR3complex is depicted in the middle. Neo-1 and Neo-4 are represented with a silver cartoon model, and TLR3 is shown in a light green surface model. The interacting residues of Neo-1 and Neo-4 with TLR3 receptor are shown on either side. Hydrogen bonds are indicated in blue lines. The color of interacting residues reflects the properties of amino acid.

Interaction analysis revealed strong molecular contacts between the designed vaccines (Neo-1 and Neo-4) and TLR3. A total of 31 residues of the Neo-1 construct interacted through hydrogen bonds with TLR3 residues, which are crucial to maintaining complex stability. Neo-1 residues that mediated hydrogen bond interaction with TLR3 with a bond distance ≤ 2.75Å are: Thr32, Arg33, Lys118 (2×), Lys174, Pro185, Thr187, and Lys189. Besides, the constructed vaccine residues that formed a hydrogen bond with TLR3 residues with a bond distance in the range of 2.76–3Å include Arg12, Ala16, Arg33, Pro92, Ala93, Ser103, Ser107, His114 (2×), and Ser115, Pro143, Gly146 (2×), Pro163, Lys174, Gly182, Pro183 (2×), Lys189, and Gly195. With a bond distance >3Å, Neo-1 residues, such as Arg12, Lys189, and Tyr180, also showed hydrogen bonding with TLR3 residues. In addition, four residues of Neo-1, including Arg33, Lys118, Lys174, and Lys189(2×), formed salt bridges with TLR3 residues (bond distance in the range of 2.64Å to 2.76Å). Detailed interactions of Neo-1 construct with TLR3 receptor are displayed in **Figure 5** and **Table S11**.

Similarly, Neo-4 construct mediated 37 hydrogen bonds with TLR3 residues. Neo-1 residues, including, Lys71 (3×), Asp75, Lys82, Thr185, Lys276 (2×), Ala279, Lys295, Asn299, Asp324, and His415, showed hydrogen bond with TLR3 residues with a bond distance ≤ 2.75Å. Moreover, Lys74 (3×), Lys82, Ser204, Asn274 (2×), Lys276, Thr278, Lys295, Pro281, Gly282, Pro283, Asp324, Gln396, Thr401, Asn409 (2×), His412, His413, and His415 residues of Neo-1 formed a hydrogen bond with TLR3 residues with a bond distance 2.76–3Å. Neo-1 residues that showed hydrogen bonding with TLR3 with a bond distance of>3Å are Lys71, Asp324, and Gln396.

Furthermore, the Neo-4 construct showed a total of 10 salt bridges interactions with TLR3 residues through Lys71, Asp75, Lys82(2×), Lys276(2×), Lys295(2×), Asp324, and His412 residues (bond distance in the range of 2.66Å to 2.79Å). Detailed interactions of the Neo-4 construct with TLR3 receptor are displayed in **Figure 5** and **Table S12**.

### 3.15 Vaccine optimization and *in-silico* cloning

**Figure 6:**
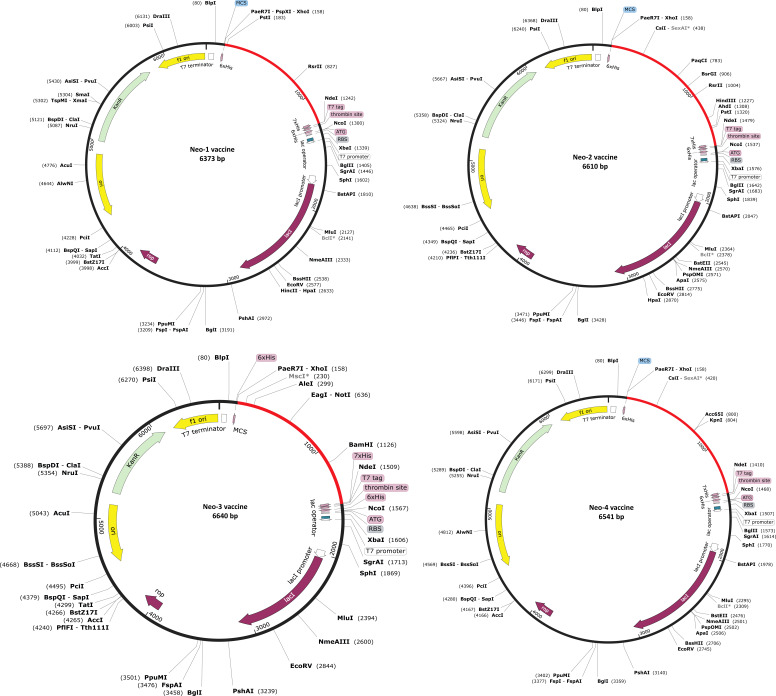
In silico restriction cloning of Neo-1−4 vaccine constructs. Using the SnapGene program, the final vaccine constructs codon-optimized sequence (red color) was cloned between the XhoI (158) and NdeI (936) restriction enzyme loci in the pET-28a (+) expression vector (black color). Efficient expression of the designed constructs can be carried out in E. coli strain K12 for effective vaccine production.

An efficient vaccine expression in the *E. coli* system is required for computational cloning (44). The prioritized B-cell and T-cell predicted epitopes and suitable adjuvants and linkers were used to create four vaccines (Neo-1–4). These vaccine sequences were submitted separately in the Backtranseq tool to modify the codon used to most sequenced prokaryotic species. The cDNA sequence of Neo-1–4 was 1089, 1326, 1428, and 1257 nucleotides, respectively, according to this analysis. The obtained CAI (Neo-1 = 0.94; Neo-2 = 0.99; Neo-3 = 0.97; Neo-4 = 0.95) showed that the modified sequences are comprised of codons capable of using the target organism’s cellular machinery. Also, the modified sequences had a GC content ranging from 50.91% to 53.76%. This sequence information indicates that the proposed vaccines will be expressed efficiently and consistently in *E. coli*. The cleavage sites for *Xho*I and *Nde*I enzymes were added at the N and C termini of Neo-1–4 cDNA (**Figure S3**), and the final optimized sequences were then inserted into the pET28a (+) vector using the SnapGene software to clone the proposed vaccines. For Neo-1–4, the cloned plasmids had final length of 6373, 6610, 6640, and 6541 bp, respectively (**Figure 6**).

### 3.16 Immunostimulatory response of the vaccine constructs

**Figure 7:**
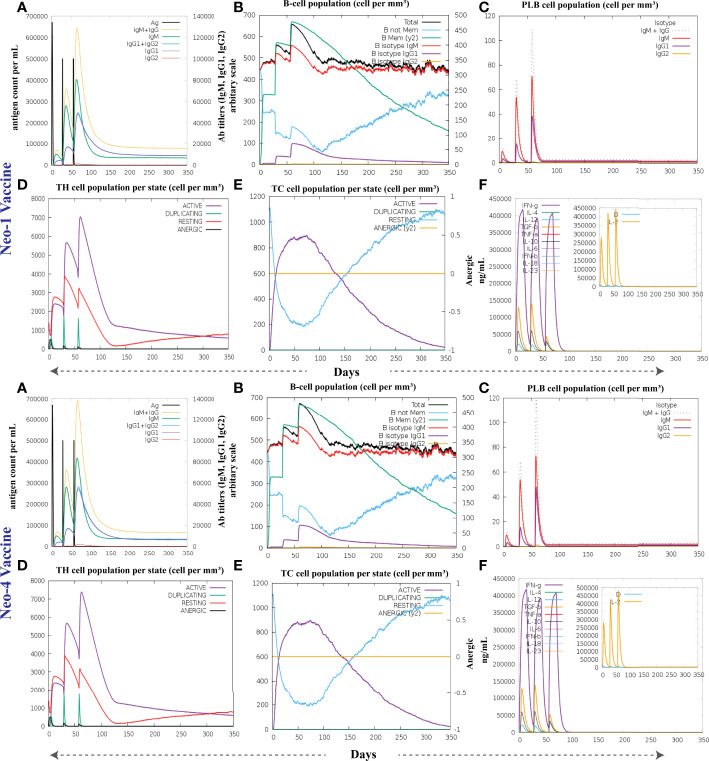
The immune response of Neo-1 and Neo-4 evaluated using C-Immsim server **(A)** Response of antibodies and antibodies complex to antigen **(B)** Total count per entity state of B-lymphocytes **(C)** Count of plasma B-lymphocytes **(D)** Total count per entity state of CD4 T helper lymphocyte **(E)** Total count per entity state of CD8 T-cytotoxic lymphocytes **(F)** cytokine concentrations and interleukin in various states shown in a smaller graph with the Simpson index shown in dotted line. In three successive immunological reactions, all units are expressed in cells/mm^3^.

To investigate the formation of adaptive immunity as well as immunological interactions, an immune simulation study was conducted for Neo-1 and Neo-4 vaccine complexes. The results of immunological simulation after three *in silico* injections of the vaccine constructs are shown in **Figure 7**, **S4 and S5**. A higher level of IgM was identified as a primary reaction in the simulation outcomes. Moreover, increased antibody activity levels were found in secondary and tertiary reactions, comprising IgG1+IgG2, IgM, and IgM+IgG antibodies. Subsequently, there was faster antigen clearance (reduced antigen load). As evidenced by higher quantities of simulated B-cells and memory B-cells growth, the vaccine elicited a substantial long-lasting immune response.

The growing rate of T-cells (including helper, cytotoxic, and regulatory T-cells) were seen, that are associated with relative immunity development. In addition, dendritic and macrophage cells showed higher concentrations, indicating good antigen presentation. Furthermore, the population of natural killer cells remained steady during immune simulation. Finally, different cytokines, notably higher quantities of the cytokine IFN-γ (over 400,000 ng/ml) were also detected. Those molecules evoke immune properties to ensure that the vaccine can succeed in human body.

### 3.17 Molecular dynamics simulation

The root means square deviation (RMSD) of the Cαatoms was estimated for two vaccine complexes. The average RMSD of the Neo-1 complex was 4.5501Å. This system experienced an increase in RMSD descriptors until the 30ns time; after that upward trend was ceased, and the system remained stable. Neo-4 complex experienced a continuous rise in RMSD until 50ns, then underwent smaller fluctuations and remained increased again toward the end of simulation. The average RMSD for this complex was 8.3117Å. Overall, there were no significant variations in both docked complexes during 110ns simulation, indicating the significance of our simulation analysis.

In order to understand changes in volume, SASA for both complexes was analyzed based on simulation trajectories. The average SASA value of Neo-1 and TLR-3 complex was 46952.58 Å, which is similar to the whole SASA profile after the 30ns. In contrast, the average SASA value for Neo-4 and TLR3 complex was 52342.26 Å. The SASA values in both simulated complexes increased initially, indicating that the volume of protein was expanded during the early phase.

The radius of gyration (Rg) of simulation trajectory gives information on the protein’s compact nature, with a greater Rg profile indicating less rigidity in the biological system. The average Rg value of the Neo-1 and Neo-4 complex was 32.9086Å and 40.4795 Å, respectively. There was an initial rise in the Rg profile of Neo-1 and TLR-3 complex. Besides, this complex Rg descriptor sustained between 32.1696 to 33.8119 Å between 0 to 110 ns, with several oscillations. This might be the cause of the system’s loose packing. The Rg value of the Neo-4 and TLR-3 complex also increased till 35ns and remained similar till the end, though there were few fluctuations.

Additionally, the root means square fluctuation (RMSF) profile was used to analyze protein flexibility across amino acid residues. The average RMSF value for Neo-1 and Neo-4 complex was 1.9823 Å and 4.1212 Å. Fluctuations of most residues in Neo-1 and TLR-3 complex were below 2.5 Å, with few residues, such as 690-720, 840-900, and 970-990, indicating larger flexibility. RMSF profile of Neo-4 and TLR3-complex revealed that most residues in this complex had RMSF profiles below 4.5 Å, with residues (especially in the region 1-250) showed larger fluctuations. These findings define the stability and stiffness of the constructed vaccines’ complex.

**Figure 8:**
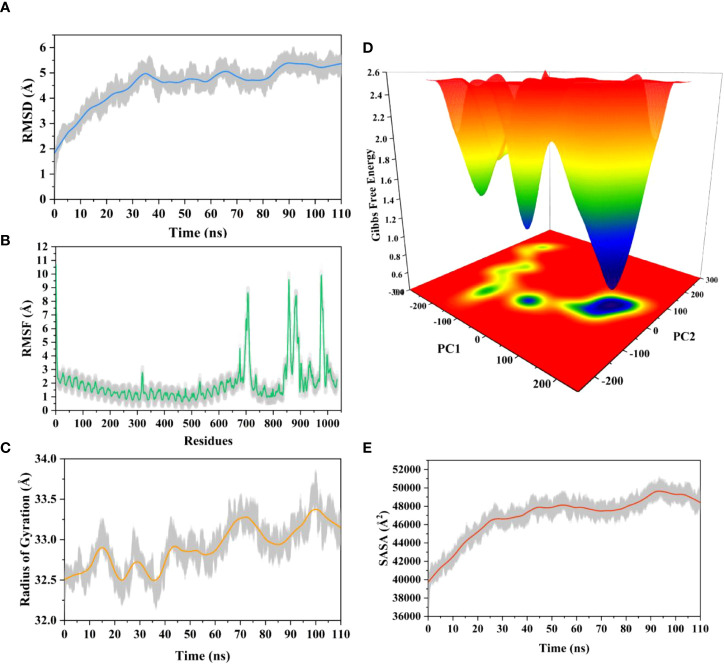
Molecular dynamics simulation of Neo-1 and TLR-3 complex at 110ns **(A)** Root means square deviation (RMSD) plot of the complex, representing mild fluctuations **(B)** The Root means square fluctuation (RMSF) plot of the docked complex **(C)** The Radius of Gyration plot of the docked complex **(D)** Free energy landscapes (FELs) of the docked complex. High, intermediate, and low/stable energy states are shown in red, yellow/green, and light-to-dark blue color in the graph, respectively **(E)** Alterations in Solvent-Accessible Surface Area (SASA) profile of the docked complex during the simulation.

**Figure 9:**
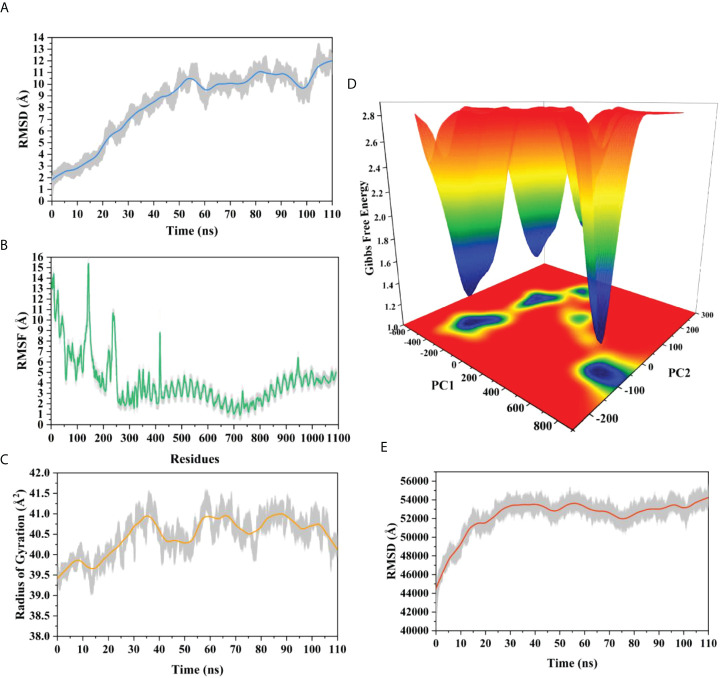
Molecular dynamics simulation of Neo-4 and TLR-3 complex at 110ns **(A)** Root means square deviation (RMSD) plot of the complex, representing mild fluctuations **(B)** The Root means square fluctuation (RMSF) plot of the docked complex **(C)** The Radius of Gyration plot of the docked complex **(D)** Free energy landscapes (FELs) of the docked complex. High, intermediate, and low/stable energy states are shown in red, yellow/green, and light-to dark blue color in the graph, respectively **(E)** Alterations in Solvent-Accessible Surface Area (SASA) profile of the docked complex during the simulation.

The transition phases of each system were analyzed by studying free energy landscape (FEL). To investigate the evolution from initial positions to metastable states, first two eigenvectors of the Neo-1- TLR-3 and Neo-4-TLR-3 complexes were employed to generate the trajectories’ FEL. To better understand the structural changes in the TLR3 upon multi-epitope vaccine binding, the low energy states in each complex were displayed. FEL plot shows the high energy, intermediate energy, and stable energy states in red, yellow, and blue, respectively. Both complexes revealed more translational conformations during the simulation. Multiple metastable states were observed in both complexes during structural changes, which is shown by high and low energy distribution. RMSD, SASA, Rg, RMSF, and FEL analysis of Neo-1/TLR3 and Neo-4/TLR3 complex is graphically presented in **Figures 8**, **9**.

During simulation, several hydrogen bonds were noted between vaccine and the TLR3 binding interface. For Neo-1 and TLR3 complex, hydrogen bonds between residues Arg12_Neo-1_ and Ala470_TLR3_ and Pro163_Neo-1_ and Lys309_TLR3_ had more than 20% occupancy during the simulation. Also, Lys189_Neo-1_ mediated multiple hydrogen bonds with Glu106 and Glu154 of TLR3 with 20% occupancy, indicating that this residue is crucial for specificity. Besides. Hydrogen bonds between residues Arg33_Neo-1_ and Glu425(2×), Thr187_Neo-1_ and Glu106, Lys174_Neo-1_ and Asp60, and Gly182_Neo-1_ and Lys179 had over 10% occupancy during the simulation. All hydrogen bond occupancy details between the Neo-1 construct and TLR3 complex is provided in **Table S13**. Similarly, for Neo-4 and TLR3 complex, higher hydrogen bonds occupancy was monitored for Gly282_Neo-4_ and Lys395 (65%) and Lys295_Neo-4_ and Asp515 (63%). In addition, the hydrogen bond between residues Lys276_Neo-4_ and Glu549, Asn299_Neo-4_ and Ser443, Lys295_Neo-4_ and Asp515 had over 45% occupancy throughout the simulation. All hydrogen bond occupancy details between Neo-4 construct and TLR3 receptor is provided in **Table S14**.

### 3.18 MM/GBSA binding free energy estimation

The binding affinity of the complex, also known as the Gibbs free energy (ΔG) in thermodynamics, is critical for predicting whether an interaction will occur in the cell under specified conditions. Thus, MM/GBSA calculation was conducted to evaluate the binding affinity of vaccine-receptor complex. The total binding energies (ΔTOTAL) of Neo-1−TLR3 and Neo-4−TLR3 complexes are -29.4841 kcal/mol and -36.7910 kcal/mol, respectively (**Table 8**). Interaction energies of both systems indicated energetically favorable and stable binding, as shown by negative Gibbs free energy values. Both Vander Waals (VDWAALS) and electrostatic energies (EEL) dominate the intermolecular interactions.

**Table 8:** MM/GBSA free energy estimation and individual free energy components of vaccines-TLR3 complexes.

Energy Component	Neo-1		Neo-4	
	Average	Std. Err. Mean	Average	Std. Err. Mean
VDWAALS	-134.5904	0.2434	-169.2386	0.3853
EEL	-719.0948	1.9689	-1076.0320	1.5889
EGB	840.8048	1.9405	1230.8375	1.6378
ESURF	-16.6036	0.0338	-22.3579	0.0517
ΔG gas	-853.6853	1.9922	-1245.2706	1.6934
ΔG solvation	824.2012	1.9315	1208.4796	1.6126
ΔTOTAL	-29.4841	0.1862	-36.7910	0.2565

## 4 Discussion

The use of immunoinformatics in the development of promising vaccines against diverse microbes, particularly viruses, are becoming more widely accepted as the first line of vaccine development (80). Epitope-based vaccines have gained much interest due to their potential benefits over traditional immunization (13). Multi-epitope vaccines have distinctive design approaches with the following characteristics, distinguishing them from conventional vaccines and single-epitope vaccines: (1) they are comprised of several MHC-restricted epitopes that T-cell receptors can identify from different T-cell subsets (2) they include CTL, HTL, and B-cell epitopes that may concurrently elicit potent cellular and humoral immune responses (3) they are made up of several epitopes from various viral antigens that can broaden the spectrum of targeted viruses (4) they introduce a few substances that can act as adjuvants, enhancing immunogenicity and producing persistent immune responses (5) they lessen undesirable substances that would otherwise cause adverse effects or abnormal immunological reactions (81). With such benefits, well-designed multi-epitope vaccines should develop into potent preventative and therapeutic treatments for viral infections. Multi-epitope vaccine for SARS-CoV-2 (13, 82) and MERS-CoV (83) have been designed using immunoinformatics-guided methods in recent years. *In silico* predicted multi-epitope vaccine was validated experimentally by *in-vitro* research against *Mycobacterium tuberculosis* (84). Recently, Yu et al. employed an immunoinformatics approach to design a multivalent vaccine against SARS-CoV-2 and its variants and confirmed vaccine immunogenicity *in vitro* and *in vivo* (85). In addition, designing a vaccine using a similar method has been shown to develop prophylactic efficiency *in vivo*, and several of these vaccines have reached the clinical trial stage (83). Considering the potential future threat associated with NeoCoV high infectivity and high transmittance rate in human population, prophylactic vaccine could be desirable. Thus, the present study aimed to exploit computational techniques to formulate multi-peptide NeoCoV vaccine candidates capable of eliciting immunological responses in possible infection of NeoCoV in humans.

Herein, NeoCoV protein components were collected to formulate a multi-epitope-based vaccine. Before antigenicity assessment of the sequences, their physical and chemical characteristics were predicted. Using the VaxiJen v2.0 virus model, the viral components were categorized as antigens and non-antigens using a cut-off of 0.4. Non-antigens were categorized as scores below this cut-off and were discarded. For appropriate component selection, the localization and transmembrane helices of the protein sequences were also assessed. This procedure determines if a prospective vaccine is suitable for experimental validation during the vaccine formulation process (86, 87). The results of all structural proteins and ORF4a, ORF4b, ORF8b, ORF5b, viral protease, NSP10, ADP Binding module, and Coronavirus endopeptidase C30 from NSPs were analyzed for epitope-based vaccine design.

In addition, these viral antigenic components were used to predict B and T-cell epitopes. A promising vaccine should be able to produce antigen-induced immunity, which is long-lasting and adaptive. Both MHC classes were expected to have a total of 44 epitopes. In contrast, the B-cells were predicted to have 19 epitopes that were immunogenic, non-toxic, and safe from causing allergic reactions. The CTL epitopes help build long-lasting immunity against viruses and infected cells (88). At the same time, HTL epitopes are linked with both humoral immunity and cell-mediated immunity. These epitopes trigger a helper T-cell response (CD4+ T cell), leading to the formation of protective memory CD8+ T cells and B-cell activation (89).

In our study, multi-epitope vaccines were constructed by fusing prioritized epitopes with appropriate adjuvants and linkers. Although safer, subunit vaccinations are often less immunogenic and effective, necessitating the use of an adjuvant. In order to enhance and direct the adaptive immune response to vaccine antigens, adjuvants are thus essential (90). Different adjuvants— β-defensin, 50S ribosomal protein L7/L12, and HBHA—were used to construct NeoCoV vaccines. By activating innate immunity cells and attracting naïve T cells through the chemokine receptor CCR-6, β-defensin peptides stimulate innate immunity cells (91). The maturation of dendritic cells, HTLs, CTLs, and IFN-γ-producing cells is induced by the 50S ribosomal protein L7/L12 when naive T-cells are stimulated (61). HBHA is a novel TLR4 agonist with minimal systemic toxicity, in addition to possessing a strong immunostimulatory potential and the potential to induce dendritic cells maturation in a TLR4-dependent manner (92). Linkers are necessary for extended conformation (flexibility), protein folding, and functional domain separation, all of which help produce a more stable protein structure (93). EAAAK linker (added after the adjuvant) was utilized to provide stiffness, minimizing any interference from other protein regions in the interaction between the adjuvant and its receptor. Following this linker, we added PADRE sequence to boost the vaccine immunogenicity. Alternatively, GGGS linker (added to separate epitope class) provides flexibility to the structure. (94). GPGPG linker (used for linking HTL epitopes) prevents junctional immunogenicity and can induce T helper cell immunological response. AAY liner (used for connecting CTL epitopes) promotes epitope presentation by assisting epitopes in forming suitable sites for binding to the TAP transporter (13). In mammals, the AAY linker is also the proteasomal cleavage site. B cell epitopes were connected by a KK linker. During the processing and presentation of epitopes through MHC-II molecules for antibody induction, the KK linker sequence is a target for the lysosomal protease enzyme (94).

ERRAT, PROCHECK, and ProSA-web servers were used to verify the predicted 3D structures of multi-epitope vaccines which showed that the overall model quality reflects X-ray crystallographic properties. Overall, the validation results suggest that the predicted vaccine structures are of high quality. Consequently, the predicted 3D structures of vaccines are reliable and can be used in downstream evaluation.

Molecular docking is vital *in silico* method to investigate pattern of interaction and affinities of ligand-receptor binding using a lock-and-key method. Protein-protein docking is often used in immunoinformatics to analyze the best, stable, and effective vaccine by examining the binding modes of proteins, interacting atoms, and binding energies. Following the same method, optimal vaccine construct(s) were predicted against immune molecules (TLR2, TLR3, and TLR4). These receptors are important in the formation of an efficient immune response, according to several immunoinformatics studies (13, 44, 95–98).TLRs are involved in both innate and adaptive immunity, as well as immune activation. The role of TLR2 and TLR4 has also been investigated in the activation of structural proteins of virus that leads to the generation of inflammatory cytokines (99). Besides, TLR3 detects viral infection and triggers an innate immunity signaling pathway (96).

The proposed constructs (Neo-1–4) were docked well with receptors (TLRs, MHC class-I and II), revealing that the designed vaccines fit well into the receptors binding pockets; hence the vaccine constructs can elicit persistent immune responses. Due to the higher docking energies and excellent performance, Neo-1 and Neo-4 were further subjected to MD simulation.

MD simulation is a robust technique to capture the motion of atoms at the atomic level, which is very difficult using experimental methods (100). The conformational stability and compactness of the vaccine-TLR3 complex were confirmed using the RMSD, RMSF, and Rg descriptors. The SASA and FEL profile of complexes revealed structural alterations induced upon the binding of the vaccine construct (ligand) with the receptor. Moreover, hydrogen bond analysis showed that hydrogen bonds were stable during the simulation and would possibly play an essential role in complex stability (101). Estimating binding free energies with MM/GBSA indicated negative ΔG scores for vaccine-TLR3 complexes; hence the values were consistent with docking scores (102). To reduce codon bias (103), computational cloning was performed on a pET28a (+) vector following codon optimization with the JCAT web service. The optimized nucleotides’ CAIs and GC content were within the acceptable limits of 0.8–1.0 and 30–70%, respectively. These results demonstrated that the proposed constructs are durable and can be expressed efficiently in *E. coli* (strain K12).

**Figure 10:**
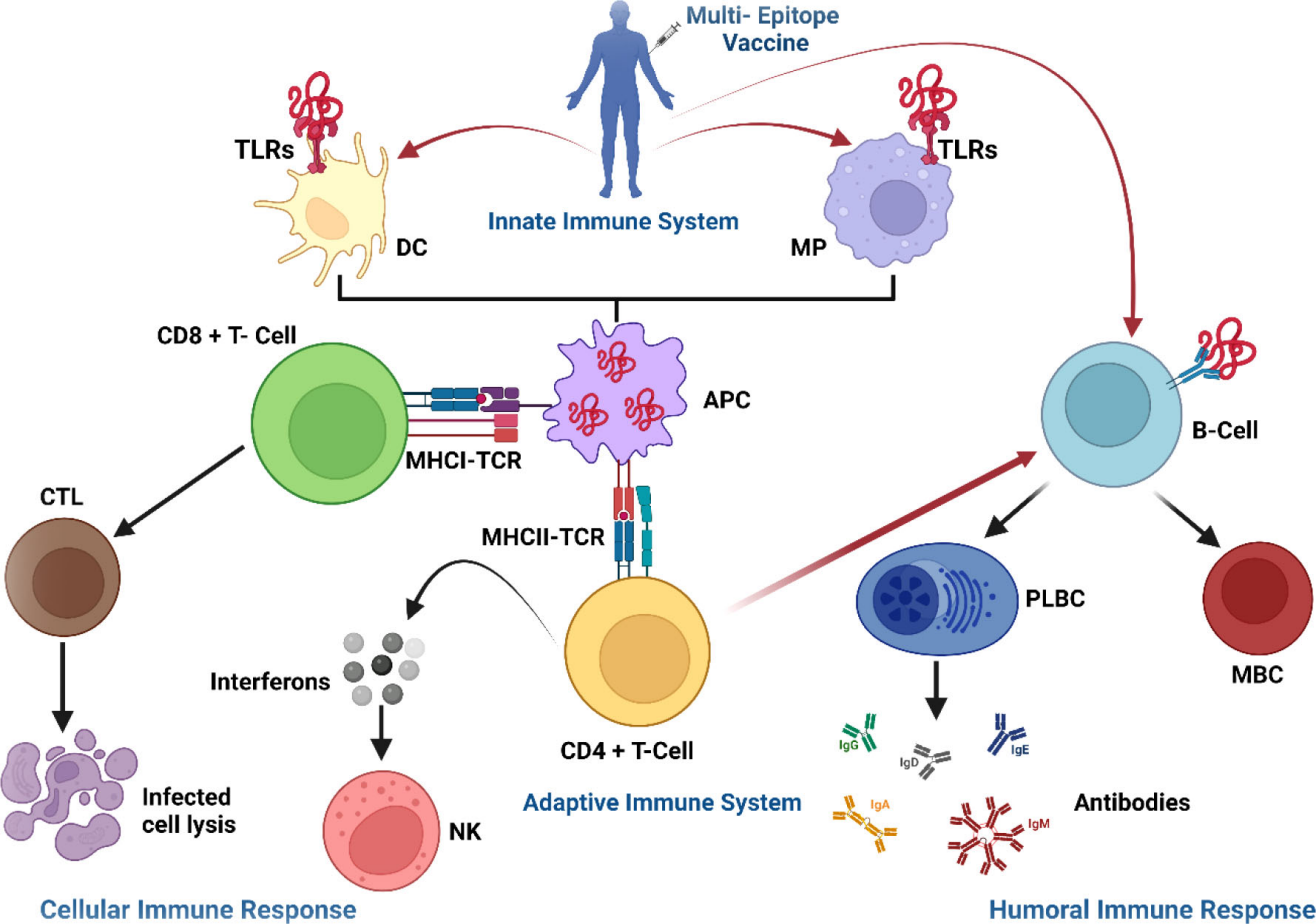
Proposed immune route in response to a multiepitope-based subunit vaccine in the host. The vaccine enters the cell through toll-like receptors (TLRs) and attaches to dendritic cells (DC) and macrophages (MP), triggering an innate immune response. The vaccine’s epitopes are then digested by antigen-presenting cells (APC) and presented to T-cells, which triggered an adaptive immunological response. T-cells activate other immune cells or destroy infected cells directly (cellular immune response), whilst plasma B-cells (PLBC) create antibodies to neutralize viruses and memory B-cells (MBC) preserve all the information needed to mount a powerful immune response in the event of re-infection (humoral immune response). TCR, T-cell receptors; CTL, cytotoxic T lymphocytes; IFNs, interferons; NK, natural killer cells; CD, cluster of differentiation; MHC, major histocompatibility complex; TCR, T-cell receptors; CTL, cytotoxic T lymphocytes; IFNs, interferons; NK, natural killer cells; PLBC, Plasma B-cells.

An immune response simulator, the C-ImmSim server, was deployed to assess the potency of the predicted vaccine to generate an immunological response (60). This method models the essential components of a functional mammalian system (bone marrow, thymus, and lymph node). The immune cells’ response to the developed vaccines, including CTL, HTL, antibodies, B-cell, dendritic cells, macrophages, and cytokines, was observed since an effective vaccine must form a lifelong adaptive immunity, hence mimic the antigen induced natural immunity. Primary antibodies (IgG and IgM), T-cell, B-cell and cytokines were triggered by immune simulation by Neo-1 and Neo-4. The proposed immune route in response to a multiepitope-based subunit vaccine (designed in this study) in the host is provided in the **Figure 10**. The developed vaccines, Neo-1 and Neo-4 may protect against NeoCoV, like previous multi-epitope vaccine design studies exploiting immunoinformatics tools (104–106). Further validation of safety and efficacy profile of the designed vaccines against NeoCoV is warranted at experimental level.

As for the NeoCoV-related viruses, various challenges for antiviral vaccines are documented. For instance, the high-affinity binding mode between MERS-CoV RBD and human DDP4 receptor required a high concentration of targeted antibodies (ribavirin) higher than what can be clinically acceptable in humans (107–109). The same is the case with SARS-CoV-2 spike protein and ACE2 receptor binding underscores the high-affinity binding requirements of the neutralizing antibodies induced by the COVID-19 vaccine (110). Another issue is the prerequisite of suitable animal models, which has hampered the vaccine safety and efficacy testing of MERS-CoV and SARS-CoV-2 disease (110, 111). Immunopathological concerns associated with subsets T cells (Th2) were raised by an earlier report regarding the MERS-CoV (110). Besides safety issues associated with the live attenuated vaccine, hypertensive lung pathology was reported for transgenic mice vaccinated with inactivated MERS-CoV vaccine (112, 113). In clinical trials, reactogenicity after vaccination and suppression of innate immune system activation is also reported for anti-SARS-CoV-2 vaccines (110). It is, however, yet to be determined whether anti-NeoCoV vaccines would demonstrate similar risks in animal models or clinical trials. On the other hand, novel vaccine development may encounter some typical challenges, including complications in the development of the production process, formulation, analytical assays, and impediments in the optimization of analytical assays. Moreover, vaccine construction can face various difficulties in low resource settings, such as technicalities, implementation of clinical trials, funding, introduction, and commercialization (114). Previously, Yan et al. demostrated that neutralizing antibodies that are elicited by the present COVID-19 vaccines against SARS-CoV-2 and MERS-CoV could not cross-neutralize the NeoCoV infection (2). Our study reports the first attempt of using comprehensive computational approach to design a multi-epitope vaccine for NeoCoV. We expect that our work could provide a starting point for the wet-lab therapeutic studies against NeoCoV infection.

## Limitation

This work presents an alternate vaccination method based on the multi-epitope assembly of NeoCoV genome protein components to address antigenic complexity. Given the limitations of our method, we were very stringent and only chose top candidate epitopes confirmed by multiple tools. Although immunoinformatics techniques were used to propose the NeoCoV vaccines, and they were predicted immunogenic, it is uncertain how much protection against NeoCoV infection they provide. This is something that future experiments will have to evaluate upon the validation of epitopes. Immunoinformatics methods are beneficial for *in silico* research and may direct laboratory investigations, saving time and money. The next step is to conduct *in vitro* immunological experiments to verify the proposed vaccines, ascertain their immunogenicity, antigenicity, safety, efficacy and develop challenge-protection preclinical trials to validate these methods.

## 5 Conclusion

Multi-epitope vaccines have already gained popularity and showed protective efficacy *in vivo*, with several undergoing clinical studies. The current study was based on an immunoinformatics-driven strategy to identify possible antigenic epitopes for inclusion in a NeoCoV vaccine candidate. Four multi-epitope vaccines were developed using three antigen categories of NeoCoV proteins including CTL, HTL, and B-cell-epitopes. Computational analysis of physicochemical and antigenic characteristics of vaccines was performed. Molecular docking and molecular dynamics simulations were used to investigate the stability profile and molecular interactions between the proposed vaccines and immunological receptors. The computer simulation showed that vaccine constructs can generate an immunological response. Several immunoinformatics methodologies were successively used to create and assess a vaccine that may provide protective immunity against viral infection; nevertheless, experimental testing is necessary to determine the precise effectiveness. The vaccine may be synthesized before being tested *in vitro* and *in vivo* in the experimental assay. We also recommend more research into the synthesis and biological activities of the multi-epitope vaccines that has been developed.

## Data availability statement

The raw data supporting the conclusions of this article will be made available by the authors, without undue reservation.

## Author contributions

SH, AK and AA-H conceived and designed the study. SA, MW and SH performed experiments. AA, AI and MI analyzed the data. SA, MW, SH and AK wrote the manuscript with inputs and comments from all co-authors. All authors contributed to the article and approved the submitted version.

## Conflict of interest

The authors declare that the research was conducted in the absence of any commercial or financial relationships that could be construed as a potential conflict of interest.

## Publisher’s note

All claims expressed in this article are solely those of the authors and do not necessarily represent those of their affiliated organizations, or those of the publisher, the editors and the reviewers. Any product that may be evaluated in this article, or claim that may be made by its manufacturer, is not guaranteed or endorsed by the publisher.

## Abbreviations

CTL, cytotoxic T lymphocytes; FEL, free energy landscape; HTL, helper T lymphocytes; MHC, major histocompatibility complex; MM-GBSA, Molecular mechanics/generalized born surface area; NeoCoV, Neo-Coronavirus; Rg, radius of gyration; RMSD, root mean square deviation; RMSF, root mean square fluctuation; SASA, solvent accessible surface area; TLR, toll-like immune receptors.
