# Supplementary material for: Exploring whole proteome to contrive multi-epitope-based vaccine for NeoCoV: An immunoinformtics and in-silico approach
Source: Front Immunol. 2022 Aug 3;13:956776. doi: 10.3389/fimmu.2022.956776 (PMC9382669; doi:10.3389/fimmu.2022.956776)
Supplement: Supplementary file 1 [file DataSheet_1.docx]

**Supporting Information**

**Exploring whole proteome to contrive multi-epitope-based vaccine for** **NeoCoV: An Immunoinformtics and *In-silico* Approach**

Shahkaar Aziz^1†^, Muhammad Waqas^2,3†^, Sobia Ahsan Halim^2†^, Amjad Ali^2^, Aqib Iqbal^1^, Maaz Iqbal^1^, Ajmal Khan^2^*, Ahmed Al-Harrasi^3^*_,_

*^1^**Institute of Biotechnology and Genetic Engineering, the University of Agriculture Peshawar, Peshawar 25000, Pakistan*

*^2^Natural and Medical Sciences Research Center, University of Nizwa, Birkat-ul-Mouz 616, Nizwa, Sultanate of Oman*

*^3^Department of Biotechnology and genetic Engineering, Haraze University Mansehra, 2100*

† = These authors equally contributed

****Correspondence:*** Ajmal Khan ([ajmalkhan@unizwa.edu.om](mailto:ajmalkhan@unizwa.edu.om)) Ahmed Al-Harrasi ([aharrasi@unizwa.edu.om](mailto:aharrasi@unizwa.edu.om))

**Table of contents**

| **Table S1.** | Retrieved NeoCoV proteins from NCBI database (NeoCoV genome ID: KC869678) |
| --- | --- |
| **Table S2.** | Prediction of MHC I binders CTL epitopes using CTLPred server with ANN and SVM cut-off of 0.51 and 0.36, respectively. |
| **Table S3*.*** | Estimated physiochemical profile and Predicted IFN-Gemma, IL4, and IL10 secretion potential of finalized MHC Class II epitopes |
| **Table S4.** | Prediction and selection of 9-mer epitopes for selected MHC II binding epitopes (15-mer peptides) with MHCPred server (threshold IC_50_ < 50). |
| **Table S*5*.** | Estimation of physiochemical properties of finalized B-cell epitopes using the Expasy ProtoParm server |
| **Table S*6*.** | Estimation of physiochemical properties of finalized B-cell epitopes using the Expasy ProtoParm server |
| **Table S*7.*** | Galaxy Refinement Scores of predicted structure models of Neo-1 to Neo-4 (Final selected model is indicated as bold letters) |
| **Table S8.** | Validation of Neo-1−4 vaccines final model structures using Ramachandran plot analysis and ERRAT overall quality score |
| **Table S9.** | Confirmational B-cell epitopes predicted for finalized 3D structure model of Neo-1 and Neo-2 vaccine construct |
| **Table S10.** | Confirmational B-cell epitopes predicted for finalized 3D structure models of Neo-3 and Neo-4 vaccine construct |
| **TableS11** | Atom-atom interactions across TLR3-Neo-1 interface obtained using PDBsum server |
| **Table S12.** | Atom-atom interactions across TLR3-Neo-4 interface obtained using PDBsum server |
| **Table S13.** | Hydrogen bonds at the TLR-3 and Neo-1 vaccine interface (with occupancy ≥ 1%) |
| **Table S14.** | Hydrogen bonds at the TLR-3 and Neo-4 vaccine interface (with occupancy ≥ 1%) |
| **Figure S1.** | Secondary structure features, relative surface accessibility, and disorder regions of constructed NeoCoV vaccines (A) Neo-2 (B) Neo-3 using NetsrurfP-3.0 server. |
| **Figure S2.** | Final modeled 3-dimensional structures representation of the vaccine construct Neo-1–4 (Blender software) |
| **Figure S3.** | The codon adapted linear vaccine constructs containing restriction sites for XhoI and NdeI, (A) Neo-1 (B) Neo-2 (C) Neo-3 (D) Neo-4 |
| **Figure S4.** | The immunostimulatory potency of the Neo-1 vaccine construct; (A) Total count of B-lymphocytes in active, internalized, and anergic states, (B) Total count of CD4 T-regulatory lymphocyte, (C) Per entity state of dendritic cell, (D) count per state of macrophages (E) population of natural killer cells. In three successive immunological reactions, all units are expressed in cells/mm^3^. |
| **Figure S5.** | The immunostimulatory potency of the Neo-4 vaccine construct; (A) Total count of B-lymphocytes in active, internalized, and anergic states, (B) Total count of CD4 T-regulatory lymphocyte, (C) Per entity state of dendritic cell, (D) count per state of macrophages (E) population of natural killer cells. In three successive immunological reactions, all units are expressed in cells/mm^3^. |

**Table S1.** Retrieved NeoCoV proteins from NCBI database (NeoCoV genome ID: KC869678)

| **Protein Name** | | **Length** | **Accession ID** |
| --- | --- | --- | --- |
| Envelope Protein (E) | | 82 | AIG13101.1 |
| Membrane Protein (M) | | 219 | AIG13102.1 |
| Nucleocapsid protein (N) | | 414 | AIG13103.1 |
| Spike Protein (S) | | 1344 | AGY29650.2 |
| ORF3 | | 103 | AGY29651.2 |
| ORF4a | | 109 | AIG13098.1 |
| ORF4b | | 258 | AIG13099.1 |
| ORF5 | | 224 | AIG13100.1 |
| ORF8b | | 199 | AIG13104.1 |
| ORF1ab Polyprotein  (7082) | NSP7 Replicase | 82 | AGR87639.3 |
|  | NSP8 Replicase | 195 |  |
|  | NSP9 Replicase | 109 |  |
|  | NSP11 | 587 |  |
|  | NSP13 | 295 |  |
|  | Nucleic acid-binding domain (NAR) | 128 |  |
|  | Viral protease | 313 |  |
|  | RNA synthesis protein NSP10 | 119 |  |
|  | Single-stranded poly (A) binding domain | 135 |  |
|  | Type III cell invasion protein SipB | 133 |  |
|  | ADP binding module | 100 |  |
|  | Coronavirus endopeptidase C30 | 277 |  |
|  | Coronavirus non-structural protein 4 C-terminus | 85 |  |
|  | Superfamily I DNA and/or RNA helicase | 242 |  |

**Table S2.** Prediction of MHC I binders CTL epitopes using CTLPred server with ANN and SVM cut-off of 0.51 and 0.36, respectively.

| **Protein** | **Epitopes** | **Score (ANN/SVM)** | **Prediction** |
| --- | --- | --- | --- |
| E | FTVVCAITL | 0.90/0.807 | Epitope |
| M | GTNSGVAIY | 0.70/1.043 | Epitope |
|  | ALSIFSAVY | 0.92/0.504 | Epitope |
|  | YPSRSMTVY | 0.85/0.810 | Epitope |
|  | LLITIVLQY | 0.52/0.471 | Epitope |
| N | STPAQNAGY | 0.66/1.006 | Epitope |
|  | SAFMGMSQF | 0.56/0.406 | Epitope |
| S | WSYTGSSFY | 0.55/1.434 | Epitope |
|  | YSTNITHLL | 0.52/0. 512 | Epitope |
|  | ISYAGAYSY | 0.58/1.349 | Epitope |
|  | MVYVITVKY | 0.69/0.361 | Epitope |
|  | FLFATVPIY | 0.61/0.431 | Epitope |
|  | SVTIADPGY | 0.84/0.842 | Epitope |
|  | ALQEVVKAL | 0.59/0.778 | Epitope |
|  | TMKKIYPAL | 0.75/0.592 | Epitope |
| ORF4b | HSPGKNLRY | 0.65/1.046 | Epitope |
|  | SVVTQPTHY | 0.92/0.463 | Epitope |

**Table S3.** Estimated physiochemical profile and Predicted IFN-ᵧ, IL4, and IL10 secretion potential of finalized MHC Class II epitopes

| **MHCII Epitopes** | **IFN-g** | **IL4** | **IL10** | **Instability index** | **Aliphatic index** | **GRAVY** | **Half-life (mammals)** | **PI** | **MW** |
| --- | --- | --- | --- | --- | --- | --- | --- | --- | --- |
| LPNEITVAKPNVLIA | +ve | inducer | Non-Inducer | 29.99 (stable) | 156.00 | 0.687 | 5.5 hours | 6 | 1591.91 |
| LIALKMVKRQSYGTN | -ve | inducer | inducer | 66 (Unstable) | 104.00 | 0.167 | 5.5 h | 10.29 | 1722.08 |
| TKSFNMVQAFGLRGA | -ve | inducer | inducer | 10.85 (stable) | 58.67 | -0.093 | 7.2 h | 11.00 | 1626.89 |
| PKVITKKDAAAAKNK | +ve | inducer | Inducer | 19.46 (stable) | 72.00 | -0.860 | >20 h | 10.18 | 1582.91 |
| SGAIKLDPKNPNYNK | +ve | inducer | Inducer | 6.22 (stable) | 58.67 | -1.420 | 1.9 h | 9.52 | 1658.87 |
| PRWYFYYTGTGPEAA | +ve | inducer | Inducer | 61.40 (Unstable) | 13.33 | -0.787 | >20h | 6.42 | 1778.94 |
| STSYYSAKPVGAYYE | +ve | inducer | Inducer | 17.25 (stable) | 32.67 | -0.660 | 1.9h | 5.72 | 1685.81 |
| PEPITTLNTRYVAPQ | +ve | inducer | Inducer | 78.00 (Unstable) | 78.00 | 6.43 | >20h | 6.43 | 1699.92 |
| ISYDIYGITGTGVFQ | +ve | inducer | Inducer | -3.18 (stable) | 97.33 | -0.500 | 20 h | 3.8 | 1633.8 |
| YVAGYKVLPPLMDVN | +ve | inducer | Non- inducer | 23.69 (stable) | 116.67 | -0.453 | 2.8 h | 5.83 | 1679.01 |
| GTQYVYSASNHKSTA | +ve | inducer | Inducer | 4.38 (stable) | 32.67 | -0.873 | 30 h | 8.50 | 1613.70 |
| TQYVYSASNHKSTAN | +ve | inducer | inducer | 10.04 (stable) | 32.6 | -1.080 | 7.2 h | 8.20 | 1670.76 |
| IIGFHSDDGNYYCVA | +ve | inducer | Non-inducer | 6.87 (stable) | 78.00 | -0.160 | 20 h | 4.20 | 1673.82 |
| TAKYTPAPGTSLHPV | +ve | inducer | Non-inducer | 34.61(stable) | 58.67 | 0.327 | 7.2 h | 8.29 | 1539.75 |
| ARDISPIAVFLRNVR | +ve | inducer | Inducer | 59.69 (Unstable) | 130.00 | -0.313 | 4.4 h | 11.70 | 1727.04 |
| STVFVPATRDSVPLH | +ve | inducer | Inducer | 60.69 (Unstable) | 90.67 | -0.240 | 1.9 h | 6.46 | 1625.85 |
| SPDFVAFNVFHGMET | +ve | inducer | Inducer | 21.66 (stable) | 45.33 | -0.220 | 1.9 h | 4.35 | 1697.88 |
| FRTVVLNNKNSYRSQ | +ve | inducer | Inducer | 55.98 (Unstable) | 64.67 | -1.033 | 1.1 h | 11.00 | 1826.05 |
| KGKFVQIPSQCTRDP | +ve | inducer | Inducer | 38.48 (stable) | 45.33 | -0.927 | 1.3 h | 9.31 | 1703.98 |
| GTGIAISVKPESTAD | +ve | inducer | Inducer | 4.17 (stable) | 84.67 | -0.033 | 30 h | 4.37 | 1445.5 |
| SKCYRAMNAYPLVVT | -ve | Inducer | Inducer | 0.63 (stable) | 78.00 | -0.173 | 1.9 h | 9.19 | 1716.05 |
| AKNILHVVGPDARAK | +ve | inducer | Non-inducer | 40.78 (stable) | 110.67 | -0.160 | 4.4 h | 9.99 | 1588.87 |
| QQLYTGFQGKQILGS | +ve | Non- inducer | inducer | 33.82 (stable) | 78.00 | -0.467 | 0.8 h | 8.59 | 1667.88 |
| TGTFTVIMRPNYTIK | +ve | Inducer | Non-inducer | 2.73 (stable) | 71.33 | -0.007 | 7.2 h | 9.99 | 1742.07 |

**Table S4.** Prediction and selection of 9-mer epitopes for selected MHC II binding epitopes (15-mer peptides) with MHCPred server (threshold IC_50_ < 50).

| **Protein** | **MHCII Epitopes** | **9-mer peptide** | **IC_50_ value** |
| --- | --- | --- | --- |
| M | LPNEITVAKPNVLIA | ITVAKPNVL | 4.38 |
|  | LIALKMVKRQSYGTN | ALKMVKRQS | 39.72 |
| N | TKSFNMVQAFGLRGA | FNMVQAFGL | 3.66 |
|  | PKVITKKDAAAAKNK | ITKKDAAAA | 6.10 |
|  | SGAIKLDPKNPNYNK | KLDPKNPNY | 8.28 |
|  | PRWYFYYTGTGPEAA | YYTGTGPEA | 3.08 |
| S | STSYYSAKPVGAYYE | YYSAKPVGA | 2.13 |
|  | PEPITTLNTRYVAPQ | PITTLNTRY | 2.10 |
|  | ISYDIYGITGTGVFQ | GITGTGVFQ | 43.15 |
|  | YVAGYKVLPPLMDVN | GYKVLPPLM | 5.90 |
|  | GTQYVYSASNHKSTA | QYVYSASNH | 2.58 |
|  | TQYVYSASNHKSTAN | QYVYSASNH | 2.58 |
|  | IIGFHSDDGNYYCVA | FHSDDGNYY | 11.61 |
| ORF4a | TAKYTPAPGTSLHPV | PAPGTSLHP | 8.2 |
| ORF4b | ARDISPIAVFLRNVR | DISPIAVFL | 1.85 |
| ORF5b | STVFVPATRDSVPLH | FVPATRDSV | 2.65 |
| PLPro | SPDFVAFNVFHGMET | PDFVAFNVF | 11.78 |
|  | FRTVVLNNKNSYRSQ | FRTVVLNNK | 2.41 |
| NSP10 | KGKFVQIPSQCTRDP | QIPSQCTRD | 14.59 |
|  | GTGIAISVKPESTAD | SVKPESTAD | 12.02 |
| ADP Binding module | SKCYRAMNAYPLVVT | YRAMNAYPL | 6.5 |
|  | AKNILHVVGPDARAK | HVVGPDARA | 11.32 |
| CoV endop.  eptidase C30. | QQLYTGFQGKQILGS | LYTGFQGKQ | 12.27 |
|  | TGTFTVIMRPNYTIK | FTVIMRPNY | 9.86 |

**Table S5.** Estimation of physiochemical properties of finalized B-cell epitopes using the Expasy ProtoParm server

| **B-cell epitopes** | **Instability index** | **Aliphatic index** | **GRAVY** | **Half-life (mammals)** | **PI** | **MW** |
| --- | --- | --- | --- | --- | --- | --- |
| TGRSVYVKFQESKPPLPPEE | 91.38 (Unstable) | 48.50 | -1.085 | 7.2 h | 5.90 | 2288.59 |
| MSNMTQLSEQQIIAIIKDWN | 57.08 (Unstable) | 102.50 | -0.210 | 30 h | 4.37 | 2363.73 |
| GNSSRGASPGPSGVGAPGGD | 54.94 (Unstable) | 24.50 | -0.725 | 30 h | 5.84 | 1683.71 |
| TNQPRGRGRNPKPRAAPNTT | -1.84 (stable) | 10.00 | -2.080 | 7.2 h | 12.48 | 2189.42 |
| PKKEKKQKAPKEESNDQEMA | 72.70 (Unstable) | 10.00 | -2.495 | >20 h | 8.72 | 2343.64 |
| EEGATDAPSTFGTRNPNNDS | 57.10 (Unstable) | 10.00 | -1.515 | 1 h | 3.92 | 2080.07 |
| NNLPPPLLSNSTGTDFKDEL | 18.61 (stable) | 78.00 | -0.755 | 1.4 h | 4.03 | 2172.38 |
| ANAKIVTLPGNDATGYCPSV | 12.92 (stable) | 83.00 | -0.135 | 4.4 h | 5.87 | 1991.25 |
| NSPTTGQLWAYNFGGVPYRV | 31.38 (stable) | 53.50 | -0.415 | 1.4 h | 8.59 | 2227.46 |
| DLGTQYVYSASNHKSTANDA | 5.79 (stable) | 49.00 | -0.900 | 1.1 h | 5.21 | 2142.22 |
| NYGATNKDDVVKPGGRASQQ | 25.55 (stable) | 39.00 | -1.345 | 1.4 h | 8.50 | 2105.25 |
| LLQPRTESKCPGNSNYVSYF | 56.31 (Unstable) | 53.50 | -0.730 | 5.5 h | 8.18 | 2303.57 |
| ESVKTPQTVPLTTGFGGEFN | 35.10 (stable) | 48.50 | -0.405 | 1 h | 4.53 | 2109.32 |
| LVSMTNHSFSVQKHVGAPAN | 11.32 (stable) | 73.00 | -0.060 | 5.5 h | 8.76 | 2124.40 |
| LGIGGDRTERLTQEMELSNW | 17.96 (stable) | 78.00 | -0.825 | 5.5 h | 4.41 | 2305.5 |
| KPVQLVPVSPVDHGGESNDS | 75.18 (Unstable) | 77.50 | -0.600 | 1.3 h | 4.54 | 2061.24 |
| KFDSGTLSKASDWKCKVTDV | 12.56 (stable) | 53.50 | -0.595 | 1.3 h | 8.16 | 2215.51 |
| FFNGADISDTIPDEKQHGCS | -12.57 (stable) | 44.00 | -0.695 | 1.1 h | 4.22 | 2181.32 |
| GAVQQESDEYILTRGPLQVG | 47.82 (stable) | 92.50 | -0.440 | 30 h | 4.14 | 2160.37 |

**Table S6**. Estimation of physiochemical properties of finalized B-cell epitopes using the Expasy ProtoParm server

| **MHCI Epitopes** | **Instability index** | **Aliphatic index** | **GRAVY** | **Half-life (mammals)** | **PI** | **MW** |
| --- | --- | --- | --- | --- | --- | --- |
| FTVVCAITL | 8.89 (stable) | 162.22 | 2.489 | 1.1 h | 5.52 | 966.20 |
| GTNSGVAIY | -17.24 (stable) | 86.67 | -0.378 | 30 h | 5.52 | 880.95 |
| ALSIFSAVY | 0.51 (stable) | 1.778 | -1.778 | 4.4 h | 5.57 | 970.13 |
| YPSRSMTVY | 102.64 (Unstable) | 32.22 | -0.544 | 2.8 h | 8.59 | 1103.26 |
| LLITIVLQY | 27.30 (stable) | 248.89 | 2.122 | 5.5 h | 5.52 | 1075.36 |
| STPAQNAGY | 20.86 (stable) | 22.22 | -0.911 | 1.9 h | 5.24 | 907.94 |
| SAFMGMSQF | 70.73 (Unstable) | 11.11 | -0.633 | 1.9 h | 5.24 | 1005.17 |
| WSYTGSSFY | -0.467 (stable) | − | -0.467 | 2.8 h | 5.52 | 1097.15 |
| YSTNITHLL | 41.01 (stable) | 130.00 | -0.211 | 2.8 h | 9.6 | 1061.20 |
| ISYAGAYSY | 25.77 (stable) | 65.56 | -0.244 | 20 h | 5.52 | 994.07 |
| SVTIADPGY | -19.41 (stable) | 86.67 | -0.244 | 1.9 h | 3.80 | 922.00 |
| ALQEVVKAL | 63.31 (Unstable) | 173.33 | -0.967 | 4.4 h | 6.05 | 970.18 |
| TMKKIYPAL | 34.57 (stable) | 97.78 | -0.067 | 7.2 h | 9.70 | 1064.35 |
| MVYVITVKY | -2.69 (stable) | 140.00 | 1.311 | 30 h | 8.25 | 1115.40 |
| FLFATVPIY | 30.29 (stable) | 130.0 | 5.52 | 1.1 h | 5.52 | 1070.30 |
| HSPGKNLRY | 61.30 (Unstable) | 43.33 | -1.711 | 3.5 h | 9.99 | 1071.20 |
| SVVTQPTHY | 61.30 (Unstable) | 64.44 | -0.378 | 1.9 h | 6.46 | 1031.1 |
| ASFSVLACY | 57.71 (Unstable) | 97.78 | -1.556 | 4.4 h | 5.56 | 960.11 |

**Table S7.** Galaxy Refinement Scores of predicted structure models of Neo-1 to Neo-4 (Final selected model is indicated as bold letters)

| **Vaccine** | **Model** | **GDT-HA** | **RMSD** | **MolProbity** | **Clash score** | **Poor Rotamers** | **Rama favored** |
| --- | --- | --- | --- | --- | --- | --- | --- |
| **Neo-1** | Initial | 1.0000 | 0.000 | 1.823 | 5.1 | 0.0 | 89.9 |
|  | **MODEL 1** | 0.9819 | 0.316 | 2.144 | 14.4 | 0.7 | 92.2 |
|  | MODEL 2 | 0.9791 | 0.311 | 2.164 | 14.7 | 0.7 | 91.9 |
|  | MODEL 3 | 0.9763 | 0.326 | 2.217 | 16.4 | 0.4 | 91.6 |
|  | MODEL 4 | 0.9805 | 0.313 | 2.159 | 14.6 | 0.4 | 91.9 |
|  | MODEL 5 | 0.9791 | 0.308 | 2.162 | 14.0 | 0.7 | 91.3 |
| **Neo-2** | Initial | 1.0000 | 0.000 | 1.131 | 0.8 | 0.0 | 94.3 |
|  | **MODEL 1** | 0.9817 | 0.314 | 1.500 | 6.5 | 0.6 | 97.2 |
|  | MODEL 2 | 0.9823 | 0.326 | 1.659 | 7.2 | 0.6 | 96.1 |
|  | MODEL 3 | 0.9692 | 0.354 | 1.600 | 6.8 | 0.0 | 96.6 |
|  | MODEL 4 | 0.9680 | 0.365 | 1.605 | 6.5 | 0.3 | 96.3 |
|  | MODEL 5 | 0.9737 | 0.348 | 1.547 | 5.9 | 0.0 | 96.6 |
| **Neo-3** | Initial | 1.0000 | 0.000 | 1.284 | 1.9 | 0.3 | 95.3 |
|  | MODEL 1 | 0.9933 | 0.245 | 1.683 | 9.3 | 0.3 | 96.9 |
|  | MODEL 2 | 0.9950 | 0.257 | 1.722 | 11.0 | 0.6 | 97.1 |
|  | MODEL 3 | 0.9911 | 0.267 | 1.725 | 9.7 | 0.3 | 96.6 |
|  | **MODEL 4** | 0.9927 | 0.266 | 1.693 | 8.4 | 0.6 | 96.4 |
|  | MODEL 5 | 0.9905 | 0.261 | 1.677 | 9.2 | 0.6 | 96.9 |
| **Neo-4** | Initial | 1.0000 | 0.000 | 1.622 | 3.1 | 0.0 | 90.8 |
|  | **MODEL 1** | 0.9873 | 0.285 | 1.956 | 11.2 | 0.6 | 94.2 |
|  | MODEL 2 | 0.9910 | 0.267 | 1.979 | 11.5 | 0.6 | 93.9 |
|  | MODEL 3 | 0.9867 | 0.282 | 1.996 | 11.3 | 0.3 | 93.5 |
|  | MODEL 4 | 0.9934 | 0.271 | 2.040 | 13.4 | 0.3 | 93.9 |
|  | MODEL 5 | 0.9892 | 0.283 | 2.010 | 12.4 | 0.0 | 93.9 |

**Table S8.** Validation of Neo-1−4 vaccines final model structures using Ramachandran plot analysis and ERRAT overall quality score

| Vaccine | Ramachandran Plot (PROCHECK) | | | | ERRAT  Quality Score |
| --- | --- | --- | --- | --- | --- |
|  | Residues in most favoured regions | Residues in additional allowed regions | Residues in generously allowed regions | Residues in disallowed regions |  |
| Neo-1 | 81.6% | 13% | 1.4% | 4% | 81.26 |
| Neo-2 | 92.3% | 6.6% | 0.6% | 0.6% | 95.87 |
| Neo-3 | 90.8% | 7.8% | 0.5% | 0.8% | 97.18 |
| Neo-4 | 85.5% | 11.5% | 1.2% | 1.8% | 90.41 |

| Vaccine | No. | Residues | No. of residues | Score |
| --- | --- | --- | --- | --- |
| Neo-1 | 1 | A:G1, A:I2, A:I3, A:N4, A:L6, A:Y9, A:Y10, A:C11, A:R12, A:R14, A:C15, A:A16, A:V17, A:L18, A:S19, A:C20, A:L21, A:P22, A:K23, A:E25, A:Q26, A:I27, A:G28, A:K29, A:C30, A:S31, A:T32, A:G34, A:R35, A:K36, A:C37, A:C38, A:R39, A:R40, A:K41, A:K42, A:E43, A:N80 | 38 | 0.796 |
|  | 2 | A:T168, A:Y170, A:S172, A:A173, A:K174, A:P175, A:V176, A:G177, A:A178, A:Y179, A:Y180, A:E181, A:G182, A:P183, A:G184, A:P185, A:G186, A:T187, A:A188, A:K189, A:Y190, A:T191, A:P192, A:A193, A:P194, A:G195, A:T196, A:S197, A:L198, A:H199, A:P200, A:V201, A:G202, A:P203, A:G204, A:P205, A:G206, A:S207, A:T208, A:V209, A:F210, A:V211, A:P212, A:A213, A:T214, A:D216, A:S217, A:V218, A:P219, A:G224, A:P225, A:G226, A:S227, A:P228, A:D229, A:F230, A:V231 | 57 | 0.714 |
|  | 3 | A:A45, A:A46, A:E256, A:S257, A:K258, A:P259, A:P260, A:L261, A:P262, A:P263, A:E264, A:E265, A:K266, A:K267, A:K289, A:G290, A:N291, A:S292, A:S293, A:R294, A:G295, A:A296, A:S297, A:P298, A:G299, A:P300, A:S301, A:G302, A:V303, A:G304, A:A305, A:P306, A:G307, A:G308, A:D309, A:K310, A:K311, A:N312, A:N313, A:L314, A:P315, A:P316, A:P317, A:L318, A:L319, A:S320, A:N321, A:S322, A:T323, A:G324, A:T325, A:D326, A:F327, A:K328, A:D329, A:E330, A:K332, A:K333, A:L334, A:G335, A:I336, A:G337, A:G338, A:D339, A:R340, A:T341, A:E342, A:T345, A:Q346, A:E349 | 70 | 0.704 |
|  | 4 | A:Y110, A:Y113, A:H114 | 3 | 0.601 |
|  | 5 | A:S115, A:P116, A:G117, A:K118, A:N119, A:R121 | 6 | 0.571 |
|  | 6 | A:T246, A:G247, A:W353, A:H357 | 4 | 0.562 |
|  | 7 | A:E240, A:G242, A:G243, A:G244, A:S245 | 5 | 0.562 |
| Neo-2 | 1 | A:R426, A:A427, A:A428, A:P429, A:N430, A:T431, A:T432, A:H433, A:H434, A:H435, A:H436 | 11 | 0.98 |
|  | 2 | A:D336, A:H337, A:G338, A:G339, A:E340, A:S341, A:N342, A:D343, A:S344, A:K345, A:K346, A:K347, A:F348, A:D349, A:S350, A:G351, A:T352, A:L353, A:S354, A:K355, A:A356, A:S357, A:D358, A:W359 | 24 | 0.807 |
|  | 3 | A:M1, A:A2, A:K3, A:L4, A:S5, A:T6, A:D7, A:E8, A:L9, A:L10, A:K11, A:M13, A:T14, A:L15, A:L16, A:E17, A:L18, A:S19, A:D20, A:F21, A:V22, A:K23, A:K24, A:F25, A:E26, A:E27, A:T28, A:F29, A:E30, A:V31, A:T32, A:A33, A:A34, A:A35, A:P36, A:V37, A:A38, A:V39, A:A40, A:A41, A:A42, :G43, A:A44, A:A45, A:P46, A:A49, A:E52 | 47 | 0.772 |
|  | 4 | A:P306, A:E307, A:P308, A:I309, A:T310, A:T311, A:L312, A:N313, A:T314, A:R315, A:Y316, A:V317, A:A318, A:P319, A:Q320, A:G321, A:G322, A:G323, A:S324, A:K325, A:P326, A:V327, A:Q328, A:L329, A:V330, A:P331, A:V332, A:H404, A:V405, A:G406, :A407, :P408, :A409, :N410, :K412, A:T413, A:N414, A:Q415, A:P416, A:R417, A:G418, A:R419, A:G420, A:R421, A:N422, A:P423, A:K424, A:P425 | 48 | 0.739 |
|  | 5 | A:S58, A:F60, A:L64, A:E65, A:A66, A:A67, A:G68, A:D69, A:K70, A:K71, A:I72, A:G73, A:V74, A:I75, A:K76, A:V77, A:V78, A:R79, A;E80, A:I81, A:V82, A:S83, A:G84, A:L85, A:G86, A:L87, A:K88, A:E89, A:A90, A:K91, A:D92, A:L93, A:V94, A:D95, A:G96, A:A97, A:P98, A:K99, A:V105, A:A106, A:K107, A:E108, A:A109, A:A110, A:D111, A:E112, A:A113, A:K114, A:A115, A:K116, A:L117, A:E118, A:A119, A:A120, A:G121, A:A122, A:T123, A:V124, A:T125, A:V126, A:K127 | 61 | 0.67 |
|  | 6 | A:Q275, A:S276, A:G278, A:T279, A:N280, A:G281, A:P282, A:G283, A:P284, A:G285, A:P286, A:K287, A:V288 | 13 | 0.65 |
|  | 7 | A:H397, A: S400, A:V401, A:Q402, A:K403 | 5 | 0.589 |
|  | 8 | A:G207, A:G208, A:K211 | 3 | 0.526 |

**Table S9.** Confirmational B-cell epitopes predicted for finalized 3D structure model of Neo-1 and Neo-2 vaccine construct

**Table S10**. Conformational B-cell epitopes predicted for finalized 3D structure models of Neo-3 and Neo-4 vaccine construct

| **Vaccine** | **No.** | **Residues** | **No. of residues** | **Score** |
| --- | --- | --- | --- | --- |
| **Neo-3** | 1 | A:L61, A:P62, A:E63, A:Q64, A:L151, A:G153, A:I154, A:E155, A:L156, A:E157, A:A158, A:A159, A:A160, A:K161, A:A162, A:K163, A:F164, A:V165, A:A166, A:A167, A:W168, A:T169, A:L170, A:K171, A:A172, A:A173, A:A174, A:G175, A:G176, A:G177, A:S178, A:L179, A:L180, A:I181, A:T182, A:I183, A:V184, A:L185, A:Q186, A:Y187, A:A188, A:A189, A:Y190, A:I191, A:Y193 | 45 | 0.817 |
|  | 2 | A:M1, A:A2, A:E3, A:N4, A:P5, A:N6, A:I7, A:D8, A:D9, A:L10, A:P11, A:L12, A:A13, A:A14, A:L15, A:G16, A:A17, A:A18, A:D19, A:G99, A:E100, A:A101, A:A102, A:L103, A:Q104, A:R105, A:L106, A:R107, A:S108, A:Q109, A:T110, A:A111, A:F112, A:E113, A:D114, A:A115, A:A117, A:R118, A:R441, A:V442, A:H443, A:H446, A:H447 | 43 | 0.756 |
|  | 3 | A:T55, A:Q58, A:F65, A:I66, A:E67, A:L68, A:R69, A:D70, A:K71, A:F72, A:T73, A:T74, A:E75, A:E76, A:L77, A:R78, A:K79, A:A80, A:E82, A:G83, A:E86, A:A87, A:N90, A:R91, A:N93, A:E94, A:E97, A:R147 | 26 | 0.723 |
|  | 4 | A:N415, A:P416, A:N417, A:N418, A:D419, A:S420, A:K421, A:K422, A:N423, A:S424, A:H444, A:H445, A:H448 | 13 | 0.713 |
|  | 5 | A:T308, A:I309, A:K310, A:G311, A:P312, A:G313, A:P314, A:G315, A:S316, A:G317, A:A318, A:N325, A:P326, A:N327, A:Y328, A:N329, A:K330, A:G331, A:G332, A:G333, A:S334, A:P335, A:K336, A:K337, A:E338, A:K339, A:K340, A:Q341, A:K342, A:A343, A:P344, A:K345, A:E346, A:E347, A:S348, A:N349, A:Q351, A:E352, A:K356, A:I361 | 40 | 0.686 |
|  | 6 | A:G231, A:P232, A:G233, A:P234, A:G235, A:F236, A:R237, A:S249, A:Q250, A:G251, A:P252, A:G253, A:P254, A:G255, A:G256, A:T257, A:G258, A:I259, A:A260, A:V283, A:G284, A:P285, A:D286, A:A287, A:R288, A:A289, A:K290, A:G291, A:P292, A:G293, A:P294, A:G295, A:T296, A:G297, A:T298 | 35 | 0.68 |
|  | 7 | A:D368, A:A369, A:T370, A:G371, A:Y372, A:P374, A:K377, A:K378, A:F379, A:F380, A:N381, A:G382, A:A383, A:D384, A:I385, A:S386, A:D387, A:T388, A:I389, A:P390, A:D391, A:E392, A:K393, A:Q394, A:H395, A:G396, A:S398 | 27 | 0.575 |
| **Neo-4** | 1 | A:M1, A:A2, A:E3, A:N4, A:P5, A:N6, A:I7, A:D8, A:D9 | 9 | 0.973 |
|  | 2 | A:A173, A:G176, A:G177, A:S178, A:L179, A:L180, A:I181, A:T182, A:I183, A:V184, A:L185, A:Q186, A:Y187, A:A188, A:A189, A:Y190, A:I191, A:S192, A:Y193, A:A194, A:G195, A:A196, A:Y197, A:S198, A:Y199, A:A200, A:A201, A:Y202, A:S203, A:V204, A:V205, A:T206, A:Q207, A:P208, A:T209, A:H210, A:Y211, A:A212, A:A213, A:Y214, A:M215, A:V216, A:Y217, A:V218, A:I219, A:T220, A:V221, A:K222, A:Y223, A:A224, A:A225, A:F227, A:L228, A:V232, A:I234 | 55 | 0.804 |
|  | 3 | A:L10, A:P11, A:L12, A:A13, A:A14, A:L15, A:G16, A:A17, A:A18, A:D19, A:L20, A:A21, A:L22, A:A23, A:T24, A:V25, A:N26, A:D27, A:L28, A:I29, A:A30, A:N31, A:L32, A:R33, A:E34, A:R35, A:A36, A:E37, A:R40, A:G83, A:E86, A:A87, A:T89, A:N90, A:R91, A:Y92, A:N93, A:E94, A:L95, A:V96, A:E97, A:R98, A:G99, A:E100, A:A101, A:A102, A:L103, A:Q104, A:R105, A:L106, A:R107, A:S108, A:Q109, A:T110, A:A111, A:F112, A:E113, A:D114, A:A115, A:S116, A:A117, A:R118, A:A119, A:E120, A:G121, A:Y122, A:V123, A:D124, A:Q125 | 69 | 0.769 |
|  | 4 | A:S291, A:T292, A:A293, A:D294, A:G295, A:P296, A:G297, A:P298, A:G299, A:A300, A:V307, A:G308, A:A311, A:R312, A:A313, A:K314, A:G315, A:P316, A:G317, A:P318, A:G319, A:T320, A:Y331, A:T332, A:I333, A:K334, A:G335, A:P336, A:G337, A:P338, A:G339, A:S340, A:G341, A:A342, A:I343, A:K344, A:L345, A:D346, A:P347, A:K348, A:N349, A:P350, A:N351, A:Y352, A:N353, A:K354, A:G355, A:G356, A:G357, A:S358, A:P359, A:K360, A:K361, A:E362, A:K363, A:K364, A:Q365, A:K366, A:A367, A:P368, A:K369, A:E370, A:E371, A:S372, A:N373 | 65 | 0.674 |
|  | 5 | A:P389, A:G390, A:N391, A:D392, A:A393, A:T394, A:G395, A:Y396, A:G420, A:S422, A:K423, A:K424, A:E425, A:E426, A:G427, A:A428, A:T429, A:D430, A:A431 | 19 | 0.605 |
|  | 6 | A:Y464, A:H467, A:H468, A:H469, A:H472 | 5 | 0.587 |
|  | 7 | A:E48, A:R51, A:T73, A:E75, A:E76, A:R78, A:K79 | 7 | 0.577 |
|  | 8 | A:T55, A:Q58, A:E59 | 3 | 0.556 |
|  |  |  |  |  |

**Table S11**. Atom-atom interactions across TLR3-Neo-1interface obtained using PDBsum server.

| TLR3 | | | | | Neo-1 construct | | | | |  |
| --- | --- | --- | --- | --- | --- | --- | --- | --- | --- | --- |
| Atom no. | **Atom name** | **Residue name** | **Residue no.** | **Chain** | **Atom no.** | **Atom name** | **Residue name** | **Residue no.** | **Chain** | **Distance & Bond Type** |
| 1 | N | Ala | 22 | A | 13665 | O | Gly | 195 | B | 2.79-H |
| 924 | OD2 | Asp | 81 | A | 13374 | NZ | Lys | 174 | B | 2.72-H |
| 1350 | OE1 | Gln | 107 | A | 13374 | NZ | Lys | 174 | B | 2.76-H |
| 1658 | OE1 | Glu | 127 | A | 13573 | NZ | Lys | 189 | B | 2.66-H |
| 1659 | OE2 | Glu | 127 | A | 13547 | OG1 | Thr | 187 | B | 2.69-H |
| 2069 | OG1 | Thr | 151 | A | 13573 | NZ | Lys | 189 | B | 3.26-H |
| 2414 | O | Gln | 174 | A | 13465 | OH | Tyr | 180 | B | 3.03-H |
| 2435 | OE1 | Glu | 175 | A | 13573 | NZ | Lys | 189 | B | 2.76-H |
| 2832 | NZ | Lys | 200 | A | 13497 | O | Gly | 182 | B | 2.87-H |
| 2832 | NZ | Lys | 200 | A | 13528 | O | Pro | 185 | B | 2.71-H |
| 3636 | NH1 | Arg | 251 | A | 13507 | O | Pro | 183 | B | 2.83-H |
| 3637 | NH2 | Arg | 251 | A | 13507 | O | Pro | 183 | B | 2.97-H |
| 4916 | NZ | Lys | 330 | A | 13233 | O | Pro | 163 | B | 2.88-H |
| 4939 | NH1 | Arg | 331 | A | 12988 | O | Gly | 146 | B | 2.88-H |
| 4940 | NH2 | Arg | 331 | A | 12988 | O | Gly | 146 | B | 2.97-H |
| 5952 | NH2 | Arg | 394 | A | 12218 | O | Pro | 92 | B | 2.85-H |
| 6304 | NZ | Lys | 416 | A | 12956 | O | Pro | 143 | B | 2.79-H |
| 6340 | NZ | Lys | 418 | A | 12230 | O | Ala | 93 | B | 2.8-H |
| 6776 | OE1 | Glu | 446 | A | 11349 | NE | Arg | 33 | B | 2.78-H |
| 6776 | OE1 | Glu | 446 | A | 11352 | NH2 | Arg | 33 | B | 2.74-H |
| 7126 | NZ | Lys | 467 | A | 11095 | O | Ala | 16 | B | 2.76-H |
| 7187 | OE1 | Gln | 470 | A | 11334 | OG1 | Thr | 32 | B | 2.63-H |
| 7494 | NH2 | Arg | 488 | A | 12426 | OG | Ser | 107 | B | 2.82-H |
| 7517 | NH1 | Arg | 489 | A | 12373 | OG | Ser | 103 | B | 2.85-H |
| 7551 | O | Ala | 491 | A | 11023 | NH1 | Arg | 12 | B | 3.13-H |
| 7551 | O | Ala | 491 | A | 11024 | NH2 | Arg | 12 | B | 2.79-H |
| 9592 | NZ | Lys | 619 | A | 12536 | O | His | 114 | B | 2.86-H |
| 9592 | NZ | Lys | 619 | A | 12531 | ND1 | His | 114 | B | 2.96-H |
| 9923 | OE1 | Glu | 639 | A | 12582 | NZ | Lys | 118 | B | 2.72-H |
| 9924 | OE2 | Glu | 639 | A | 12582 | NZ | Lys | 118 | B | 2.64-H |
| 9989 | NH2 | Arg | 643 | A | 12547 | OG | Ser | 115 | B | 2.88-H |
| 924 | OD2 | Asp | 81 | A | 3374 | NZ | Lys | 174 | B | 2.72-Salt bridge |
| 1658 | OE1 | Glu | 127 | A | 13573 | NZ | Lys | 189 | B | 2.66-Salt bridge |
| 2435 | OE1 | Glu | 175 | A | 13573 | NZ | Lys | 189 | B | 2.76-Salt bridge |
| 6777 | OE2 | Glu | 446 | A | 11352 | NH2 | Arg | 33 | B | 2.74-Salt bridge |
| 9923 | OE1 | Glu | 639 | A | 12582 | NZ | Lys | 118 | B | 2.64-Salt bridge |
| Non-Bonded residues of Neo-1= Ile3, Arg12, Val13, Ala16, Val17, Leu18, Thr32, Arg33, Pro92, Ala93, Ala96, Gly97, Ala100, Trp102, Ser103, Tyr104, Ser107, His114, Ser115, Lys118, Gly144, Pro145, Gly146, Thr147, Pro163, Pro165, Gly166, Lys174, Tyr180, Gly182, Pro183, Gly184, Pro185, Thr187, Lys189, Tyr190, Pro192, Ala193, Pro194, Gly195, Gly206, Pro225, Phe230, Val231, Thr296 | | | | | | | | | | |

**Table S12**. Atom-atom interactions across TLR3-Neo-4 interface obtained using PDBsum server.

| TLR3 | | | | | Neo-4 | | | | |  |
| --- | --- | --- | --- | --- | --- | --- | --- | --- | --- | --- |
| Atom no. | **Atom name** | **Residue name** | **Atom no.** | **Chain** | **Atom no.** | **Atom name** | **Residue name** | **Residue no.** | **Chain** | **Distance & Bond Type** |
| 4122 | OG | Ser | 282 | A | 16754 | NE2 | Gln | 396 | B | 3.14-H |
| 4512 | OE2 | Glu | 306 | A | 16754 | NE2 | Gln | 396 | B | 2.84-H |
| 4530 | OH | Tyr | 307 | A | 16832 | OG1 | Thr | 401 | B | 2.8-H |
| 4916 | NZ | Lys | 330 | A | 15644 | OD2 | Asp | 324 | B | 2.66-H |
| 4939 | NH1 | Arg | 331 | A | 15646 | O | Asp | 324 | B | 2.79-H |
| 4940 | NH2 | Arg | 331 | A | 15646 | O | Asp | 324 | B | 3.08-H |
| 5007 | NZ | Lys | 335 | A | 16936 | OD1 | Asn | 409 | B | 2.78-H |
| 5510 | OD2 | Asp | 366 | A | 16986 | NE2 | His | 412 | B | 2.79-H |
| 5860 | ND2 | Asn | 388 | A | 16936 | OD1 | Asn | 409 | B | 2.9-H |
| 5951 | NH1 | Arg | 394 | A | 17040 | O | His | 415 | B | 2.78-H |
| 5952 | NH2 | Arg | 394 | A | 17041 | OXT | His | 415 | B | 2.69-H |
| 6304 | NZ | Lys | 416 | A | 15082 | O | Pro | 281 | B | 2.77-H |
| 6304 | NZ | Lys | 416 | A | 15093 | O | Gly | 282 | B | 2.77-H |
| 6304 | NZ | Lys | 416 | A | 15103 | O | Pro | 283 | B | 2.8-H |
| 6700 | O | Asn | 441 | A | 17003 | NE2 | His | 413 | B | 2.93-H |
| 7077 | OG | Ser | 464 | A | 15332 | OD1 | Asn | 299 | B | 2.68-H |
| 7094 | OH | Tyr | 465 | A | 15063 | O | Ala | 279 | B | 2.68-H |
| 7491 | NE | Arg | 488 | A | 15049 | OG1 | Thr | 278 | B | 2.95-H |
| 7517 | NH1 | Arg | 489 | A | 14000 | OG | Ser | 204 | B | 2.89-H |
| 8257 | OD1 | Asp | 536 | A | 15276 | NZ | Lys | 295 | B | 2.76-H |
| 8781 | OD1 | Asn | 568 | A | 15018 | NZ | Lys | 276 | B | 2.89-H |
| 8815 | OE1 | Glu | 570 | A | 15018 | NZ | Lys | 276 | B | 2.67-H |
| 8882 | OD2 | Asp | 575 | A | 13713 | OG1 | Thr | 185 | B | 2.62-H |
| 9175 | OD1 | Asp | 592 | A | 15276 | NZ | Lys | 295 | B | 2.73-H |
| 9176 | OD2 | Asp | 592 | A | 15018 | NZ | Lys | 276 | B | 2.72-H |
| 9292 | OG1 | Thr | 600 | A | 12008 | NZ | Lys | 74 | B | 2.78-H |
| 9663 | OG1 | Thr | 623 | A | 12008 | NZ | Lys | 74 | B | 2.89-H |
| 9677 | OG | Ser | 624 | A | 12008 | NZ | Lys | 74 | B | 2.83-H |
| 9707 | OE2 | Glu | 626 | A | 11943 | NZ | Lys | 71 | B | 2.67-H |
| 9744 | NZ | Lys | 628 | A | 11945 | O | Lys | 71 | B | 2.72-H |
| 9744 | NZ | Lys | 628 | A | 12028 | OD1 | Asp | 75 | B | 2.74-H |
| 9923 | OE1 | Glu | 639 | A | 14986 | ND2 | Asn | 274 | B | 2.79-H |
| 9989 | NH2 | Arg | 643 | A | 14985 | OD1 | Asn | 274 | B | 2.80-H |
| 10077 | OD1 | Asp | 648 | A | 12132 | NZ | Lys | 82 | B | 2.86-H |
| 10110 | O | Cys | 651 | A | 11943 | NZ | Lys | 71 | B | 3.27-H |
| 9707 | OE2 | Glu | 626 | A | 11943 | NZ | Lys | 71 | B | 2.67-H |
| 10120 | O | Glu | 652 | A | 11943 | NZ | Lys | 71 | B | 2.70-H |
| 10125 | OE2 | Glu | 652 | A | 12132 | NZ | Lys | 82 | B | 2.70-H |
| 4916 | NZ | Lys | 330 | A | 15643 | OD1 | Asp | 324 | B | 2.66-Salt bridge |
| 5510 | OD2 | Asp | 366 | A | 16986 | NE2 | His | 412 | B | 2.79-Salt bridge |
| 8258 | OD2 | Asp | 536 | A | 15276 | NZ | Lys | 295 | B | 2.76-Salt bridge |
| 8815 | OE1 | Glu | 570 | A | 15018 | NZ | Lys | 276 | B | 2.67-Salt bridge |
| 9176 | OD2 | Asp | 592 | A | 15018 | NZ | Lys | 276 | B | 2.72-Salt bridge |
| 9175 | OD1 | Asp | 592 | A | 15276 | NZ | Lys | 295 | B | 2.73-Salt bridge |
| 9706 | OE1 | Glu | 626 | A | 11943 | NZ | Lys | 71 | B | 2.67-Salt bridge |
| 9744 | NZ | Lys | 628 | A | 12028 | OD1 | Asp | 75 | B | 2.74-Salt bridge |
| 10078 | OD2 | Asp | 648 | A | 12132 | NZ | Lys | 82 | B | 2.71-Salt bridge |
| 10125 | OE2 | Glu | 652 | A | 12132 | NZ | Lys | 82 | B | 2.7-Salt bridge |
| Non-bonded residues of Neo-4= Lys71, Lys74, Asp75, Leu81, Lys82, Val181, Ile184, Thr185, Tyr188, Ala189, Phe192, Leu193, Thr196, Val197, Tyr200, Gly201, Ser204, Pro205, Asn274, Lys276, Thr278, Ala279, Gly280, Pro281, Gly282, Pro283, Asn293, Lys295, Asn299, Gly300, Pro301, Asp324, Thr394, Gln396, Val398, Pro399, Thr401, Phe408, Asn409, His411, His412, His413, His415 | | | | | | | | | | |

**Table S13**. Hydrogen bonds at the TLR-3 and Neo-1 vaccine interface (with occupancy ≥ 1%)

| **TLR3 residues (1-676) ----Neo-1 residues (677-1035)** | **Occupancy (%)** | **Avg. distance** | **Lifetime (Avg.)** | **Lifetime (frames)** |
| --- | --- | --- | --- | --- |
| Ala470@O **:** Arg12@NH2 | 39.5 | 2.8 | 11.8798 | 4348 |
| Lys309@NZ**:** Pro163@O | 21.5 | 2.83 | 3.8374 | 2360 |
| Glu106@OE2 **:** Lys189@NZ | 20.7 | 2.81 | 3.8124 | 2276 |
| Glu106@OE1 **:** Lys189@NZ | 20 | 2.8 | 4.5496 | 2202 |
| Glu425@OE2 **:** Arg33@NH2 | 20 | 2.83 | 3.0165 | 2196 |
| Glu106@OE2 **:** Thr187@OG1 | 18.9 | 2.69 | 23.8851 | 2078 |
| Asp60@OD2 **:** Lys174@NZ | 17.6 | 2.79 | 8.5956 | 1934 |
| Glu425@OE2 **:** Arg33@NE | 14.7 | 2.82 | 4.8675 | 1616 |
| Glu154@OE1 **:** Lys189@NZ | 13.4 | 2.79 | 7.0625 | 1469 |
| Lys179@NZ **:** Gly182@O | 10.3 | 2.83 | 4.2707 | 1136 |
| Gln449@OE1 **:** Thr32@OG1 | 9.68 | 2.69 | 23.1522 | 1065 |
| Arg310@NH2 **:** Gly146@O | 6.55 | 2.87 | 1.7433 | 720 |
| Arg467@NH2 **:** Ser107@OG | 5.39 | 2.88 | 2.6955 | 593 |
| Gln86@OE1 **:** Lys174@NZ | 4.74 | 2.84 | 3.1386 | 521 |
| Tyr447@OH **:** Arg33@O | 4.72 | 2.76 | 5.9655 | 519 |
| Lys397@CE **:** Ala93@O | 4.13 | 2.92 | 1.3009 | 454 |
| Lys179@NZ **:** Pro185@O | 2.95 | 2.84 | 3.0857 | 324 |
| Ala1@N **:** Gly195@O | 2.82 | 2.84 | 2.8182 | 310 |
| Lys395@NZ **:** Pro143@O | 2.57 | 2.83 | 3.7733 | 283 |
| Asp15@OD1 **:** Gly195@CA | 1.2 | 2.93 | 1.2222 | 132 |
| Glu106@OE2 **:** Thr187@CG2 | 1.18 | 2.94 | 1.1207 | 130 |

**Table S14.** Hydrogen bonds at the TLR-3 and Neo-4 vaccine interface (with occupancy ≥ 1%)

| **TLR3 residues (416-1096) : Neo-4 (1-415)** | **Occupancy (%)** | **Avg. distance** | **Lifetime (Avg.)** | **Lifetime (frames)** |
| --- | --- | --- | --- | --- |
| Lys395@NZ **:** Gly282@O | 65.4 | 2.82 | 6.3251 | 7198 |
| Asp515@OD1 **:** Lys295@NZ | 63.6 | 2.82 | 4.0416 | 6996 |
| Glu549@OE2 **:** Lys276@NZ | 49.6 | 2.81 | 5.0546 | 5459 |
| Ser443@OG **:** Asn299@OD1 | 48.5 | 2.73 | 8.7553 | 5332 |
| Asp515@OD2 **:** Lys295@NZ | 46.4 | 2.85 | 4.0416 | 6996 |
| Gln444@OH **:** Ala279@O | 27 | 2.82 | 1.9329 | 2965 |
| Asp571@OD2 **:** Lys276@NZ | 26.1 | 2.78 | 9.4539 | 2874 |
| Lys309@NZ **:** Asp324@OD1 | 22.2 | 2.8 | 6.2699 | 2439 |
| Glu605@OE1 **:** Leu71@NZ | 10.2 | 2.79 | 8.6538 | 1125 |
| Glu631@O **:** Leu71@NZ | 10.1 | 2.82 | 6.1611 | 1109 |
| Arg468@NH1 **:** Ser204@OG | 9.92 | 2.88 | 1.6606 | 1091 |
| Glu285@OE2 **:** Gln396@NE2 | 7.27 | 2.84 | 3.8278 | 800 |
| Asp554@OD2 **:** Thr185@OG1 | 6.75 | 2.74 | 7.5816 | 743 |
| Tyr286@OH **:** Thr401@OG1 | 6.65 | 2.8 | 4.4634 | 732 |
| Lys607@NZ **:** Asp75@OD1 | 5.67 | 2.76 | 17.3333 | 624 |
| Asp345@OD2 **:** His412@NE2 | 4.99 | 2.8 | 6.6145 | 549 |
| Asp15@OD1 **:** Lys345@NZ | 3.45 | 2.8 | 5.1351 | 380 |
| Thr602@OG1 **:** Lys74@NZ | 2.56 | 2.86 | 2.4956 | 282 |
| Thr579@OG1 **:** Lys74@NZ | 2.39 | 2.87 | 2.3909 | 263 |
| Arg373@NH2 **:** His415@OXT | 1.66 | 2.81 | 3.9783 | 183 |
| Arg373@NH1 **:** His415@O | 1.63 | 2.82 | 3.9778 | 179 |


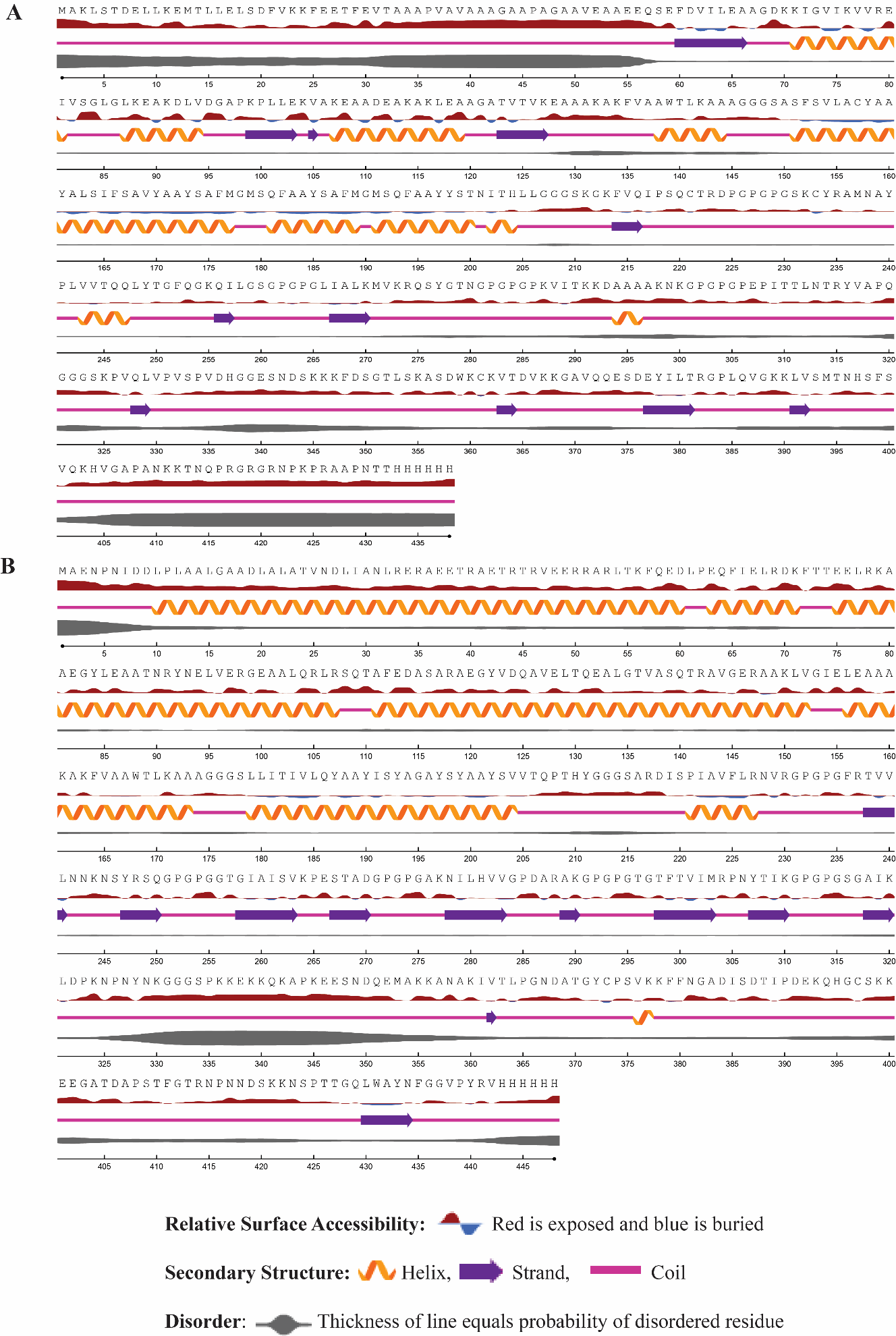


**Figure S1.** Secondary structure features, relative surface accessibility, and disorder regions of constructed NeoCoV vaccines **(A)** Neo-2 **(B)** Neo-3 using NetsrurfP-3.0 server.


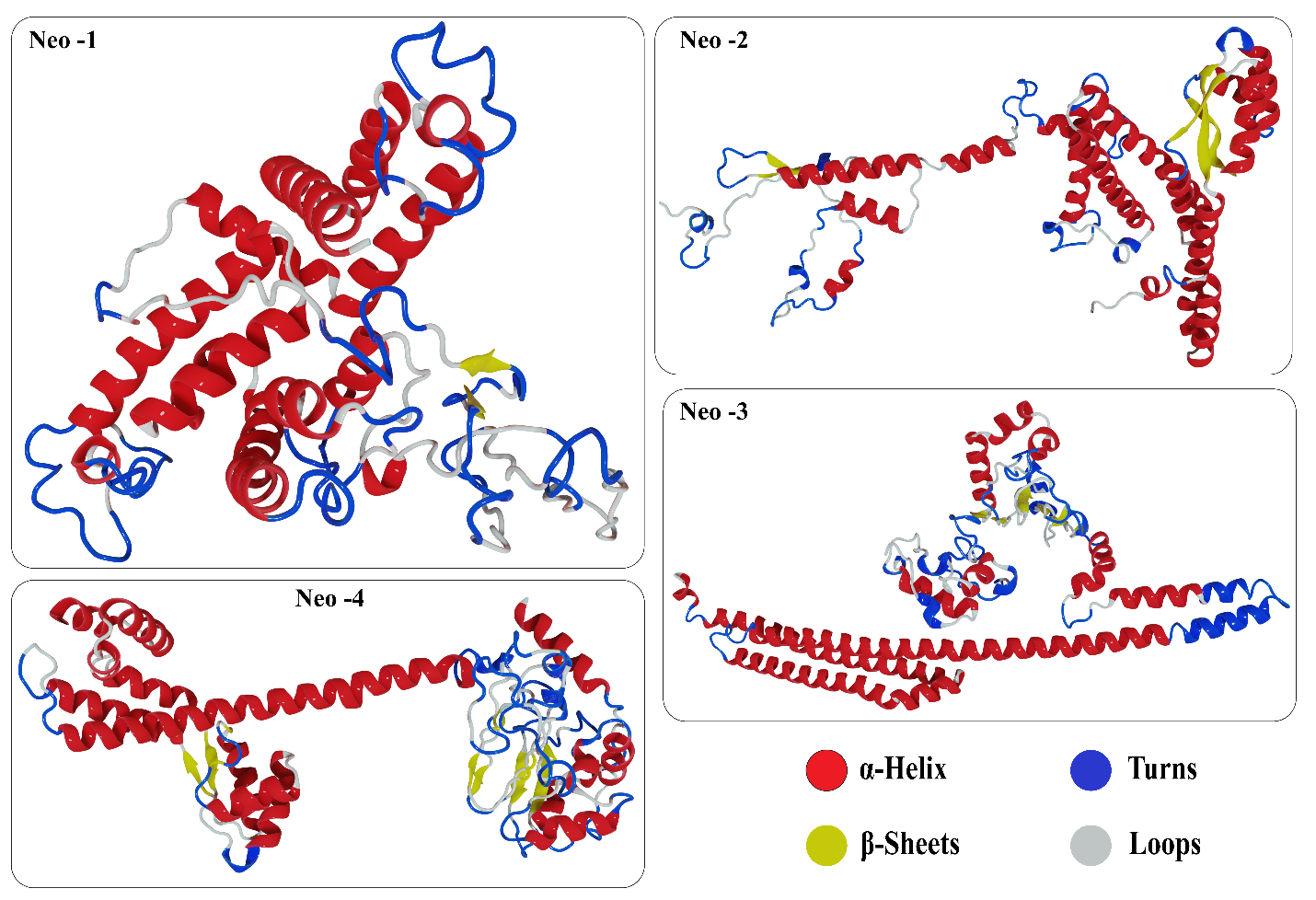


**Figure S2.** Final modeled 3-dimensional structures representation of the vaccine construct Neo-1–4 (Blender software)


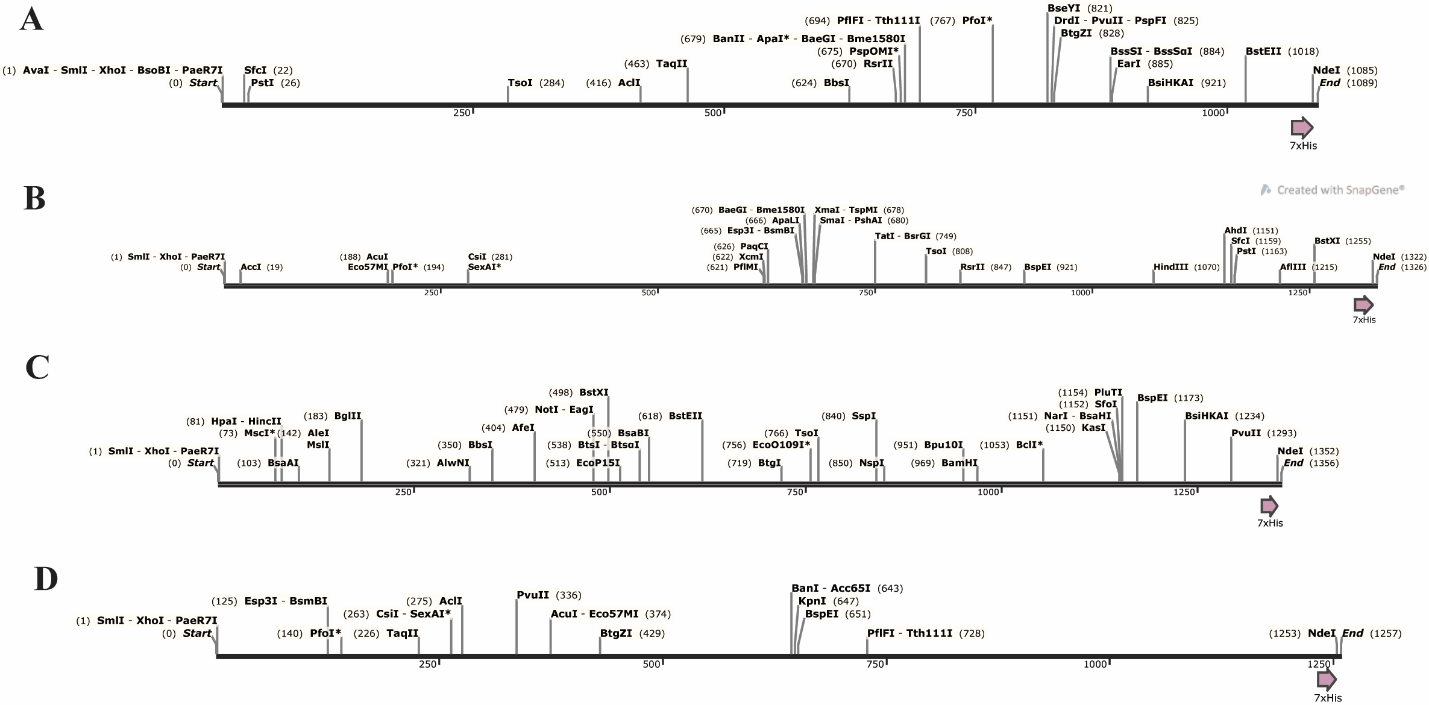


**Figure S3.** The codon adapted linear vaccine constructs containing restriction sites for XhoI and NdeI, (A) Neo-1 (B) Neo-2 (C) Neo-3 (D) Neo-4


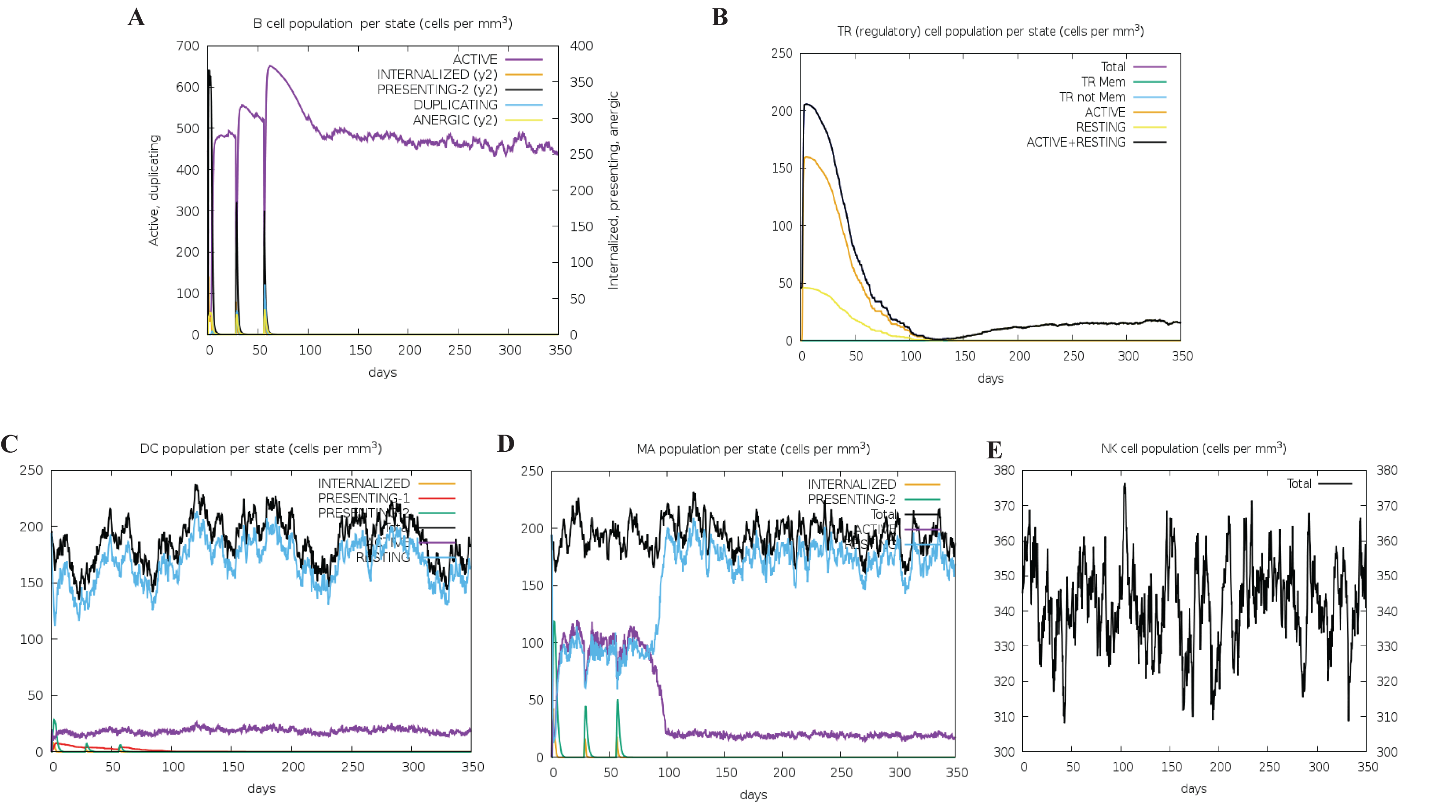


**Figure S4.** The immunostimulatory potency of the Neo-1 vaccine construct; **(A)** Total count of B-lymphocytes in active, internalized, and anergic states, **(B)** Total count of CD4 T-regulatory lymphocyte, **(C)** Per entity state of dendritic cell, **(D)** count per state of macrophages **(E)** population of natural killer cells. In three successive immunological reactions, all units are expressed in cells/mm^3^.


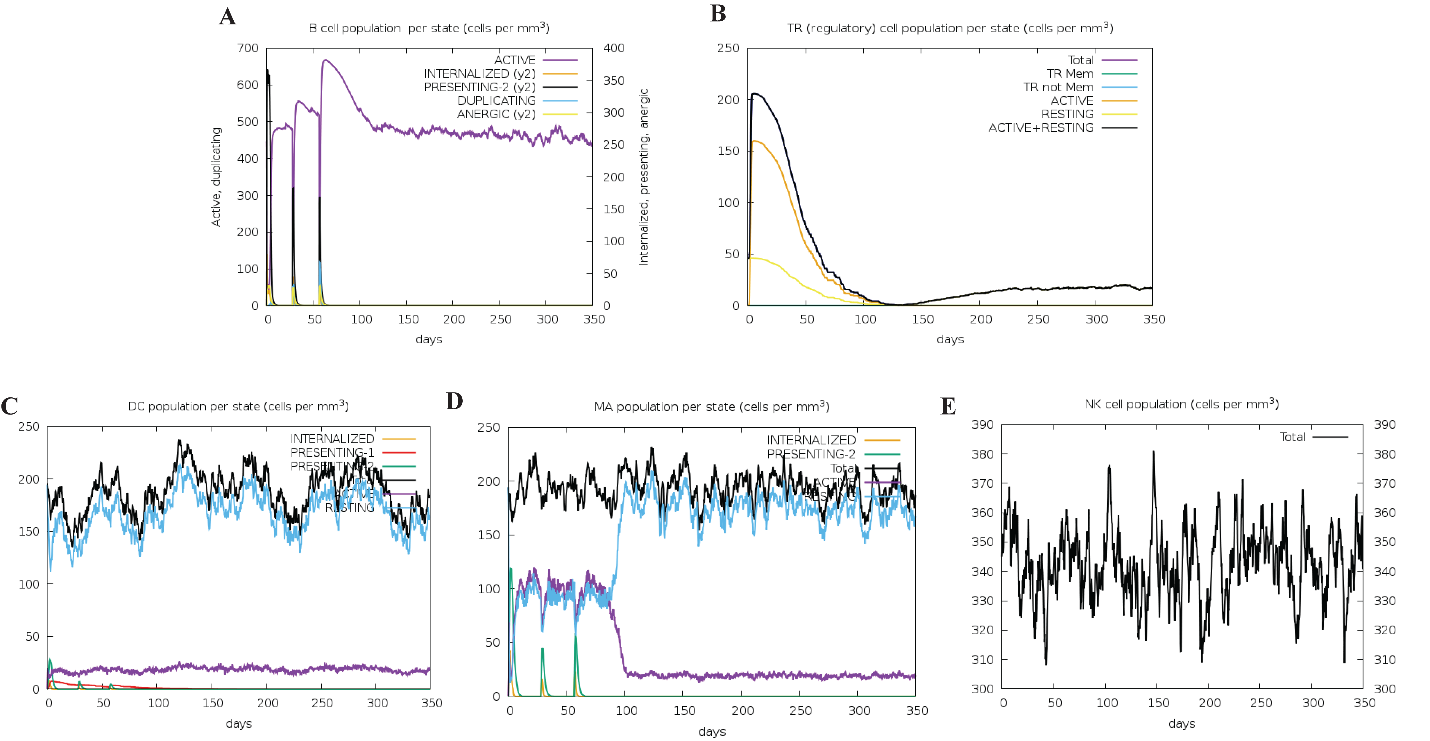


**Figure S5**. The immunostimulatory potency of the Neo-4 vaccine construct; **(A)** Total count of B-lymphocytes in active, internalized, and anergic states, **(B)** Total count of CD4 T-regulatory lymphocyte, **(C)** Per entity state of dendritic cell, **(D)** count per state of macrophages **(E)** population of natural killer cells. In three successive immunological reactions, all units are expressed in cells/mm^3^.
